# Development of a Proximity Labeling System to Map the *Chlamydia trachomatis* Inclusion Membrane

**DOI:** 10.3389/fcimb.2017.00040

**Published:** 2017-02-15

**Authors:** Elizabeth A. Rucks, Macy G. Olson, Lisa M. Jorgenson, Rekha R. Srinivasan, Scot P. Ouellette

**Affiliations:** Division of Basic Biomedical Sciences, Sanford School of Medicine, The University of South DakotaVermillion, SD, USA

**Keywords:** *Chlamydia trachomatis*, Inc, inclusion membrane, proximity labeling

## Abstract

*Chlamydia* grows within a membrane-bound vacuole termed an inclusion. The cellular processes that support the biogenesis and integrity of this pathogen-specified parasitic organelle are not understood. *Chlamydia* secretes integral membrane proteins called Incs that insert into the chlamydial inclusion membrane (IM). Incs contain at least two hydrophobic transmembrane domains flanked by termini, which vary in size and are exposed to the host cytosol. In addition, Incs are temporally expressed during the chlamydial developmental cycle. Data examining Inc function are limited because of (i) the difficulty in working with hydrophobic proteins and (ii) the inherent fragility of the IM. We hypothesize that Incs function collaboratively to maintain the integrity of the chlamydial inclusion with small Incs organizing the IM and larger Incs interfacing with host cell machinery. To study this hypothesis, we have adapted a proximity-labeling strategy using APEX2, a mutant soybean ascorbate peroxidase that biotinylates interacting and proximal proteins within minutes in the presence of H_2_O_2_ and its exogenous substrate, biotin-phenol. We successfully expressed, from an inducible background, APEX2 alone, or fusion proteins of IncA_TM_ (TM = transmembrane domain only), IncA, and IncF with APEX2 in *Chlamydia trachomatis* serovar L2. IncF-APEX2, IncA_*TM*_-APEX2, and IncA-APEX2 localized to the IM whereas APEX2, lacking a secretion signal, remained associated with the bacteria. We determined the impact of overexpression on inclusion diameter, plasmid stability, and Golgi-derived sphingomyelin acquisition. While there was an overall impact of inducing construct expression, IncF-APEX2 overexpression most negatively impacted these measurements. Importantly, Inc-APEX2 expression in the presence of biotin-phenol resulted in biotinylation of the IM. These data suggest that Inc expression is regulated to control optimal IM biogenesis. We subsequently defined lysis conditions that solubilized known Incs and were compatible with pulldown conditions. Importantly, we have created powerful tools to allow direct examination of the dynamic composition of the IM, which will provide novel insights into key interactions that promote chlamydial growth and development within the inclusion.

## Introduction

*Chlamydia* is an obligate intracellular bacterium and major pathogen of humans. *C. trachomatis* (Ctr) serovars cause either blinding trachoma (Schachter, 1999) or the most common bacterial sexually transmitted infection (STI) (Datta et al., 2007). It is estimated that 70% of primary chlamydial infections in women are asymptomatic in keeping with the ability of the pathogen to limit typical host inflammatory responses to infection (Darville and Hiltke, 2010). As a consequence, these infections may ascend from the cervix into the upper genital tract potentiating the development of pelvic inflammatory disease, ectopic pregnancy, and/or infertility. Highlighting the long-term significance of these issues are CDC estimates indicating that 10% of women between the ages of 15 and 19 will test positive for *Chlamydia* (Centers for Disease Control and Prevention, 2013). Further, the CDC calculates the cost of treating chlamydial genital infections and associated conditions in the U.S. exceeds 700 million dollars annually (Satterwhite et al., 2011).

Intracellular survival and the ability to limit host response to infection are linked to the intracellular lifestyle of *Chlamydia* and its developmental program within the confines of an intracellular vacuole, termed an inclusion. The developmental cycle is initiated when metabolically quiescent elementary bodies (EBs) are engulfed by cervical epithelial cells and differentiate within an inclusion into non-infectious, metabolically active reticulate bodies (RBs). Within the inclusion, the RBs create progeny via a newly described polarized budding mechanism (AbdelRahman et al., 2016). The completion of the developmental cycle is marked by an asynchronous differentiation of RBs into infectious EBs (see AbdelRahman and Belland, 2005 for review). The two forms of *Chlamydia* reflect their distinct roles: the EB efficiently mediates attachment and internalization into a susceptible host cell (thereby limiting exposure to professional phagocytes), and the RB, from within the inclusion, effectively orchestrates intracellular host-chlamydial interactions to efficiently harvest nutrients supporting the development of the inclusion and creation of progeny.

The inclusion membrane simultaneously functions in two distinct capacities. Firstly, it provides a barrier protecting *Chlamydia* from intracellular host cell defenses. Secondly, it creates a platform to support host-chlamydial interactions. The development of the chlamydial inclusion membrane follows the intra-inclusion progression of *Chlamydia* through the developmental cycle. While existing type III secretion effectors within the EB instigate entry into epithelial cells, proper inclusion development requires *de novo* chlamydial protein synthesis within the first 2 h of infection (Scidmore et al., 2003). Specifically, chlamydial protein synthesis is required to make newly translated type III effector proteins that function to remodel the inclusion membrane within the first few hours post-infection (Fields et al., 2003). An important class of chlamydial type III secretion effectors is classified as Incs. Incs are proteins that encode at least two transmembrane domains, which are flanked by termini that are exposed on the host cytosolic face of the chlamydial inclusion (Rockey et al., 1997; Bannantine et al., 1998; Scidmore-Carlson et al., 1999; Dehoux et al., 2011; Lutter et al., 2012). Highlighting their importance to the chlamydial intracellular lifestyle, Ctr encodes greater than 50 *inc* genes, which represents 6% of the total coding capacity of a highly reduced genome (Stephens et al., 1998). Incs are expressed and inserted into the inclusion membrane in temporally defined patterns (Shaw et al., 2000; Gauliard et al., 2015), suggesting that Incs play specific roles within the inclusion membrane at specific stages of the chlamydial developmental cycle (Moore and Ouellette, 2014).

The proposed function of Incs is articulated on a theoretical level only. Data on their specific function in the chlamydial inclusion membrane are limited due to the difficulty in working with proteins with large hydrophobic regions. In the field, it is widely accepted that Incs serve to interact with host machinery, and the limited data seemingly support this idea (Scidmore and Hackstadt, 2001; Rzomp et al., 2006; Alzhanov et al., 2009; Derre et al., 2011; Lutter et al., 2013; Dumoux et al., 2015; Kokes et al., 2015; Mital et al., 2015). Some Incs contain cytosolic domains between 60 and 200 amino acids in size, and such structured domains would certainly support interactions with eukaryotic host proteins. Other Incs contain cytosolic domains with less than 30 amino acids in size, which creates less feasible targets for eukaryotic proteins. Recent data demonstrate that specific Incs may also interact with one another and colocalize into “membrane microdomains” within the inclusion membrane (Mital and Hackstadt, 2011; Weber et al., 2015). Further, a bacterial two-hybrid (BACTH) system determined that certain Incs, like IncF, have the ability to homodimerize and also interact extensively with other Inc proteins (Gauliard et al., 2015). Inclusion engagement of specific host pathways is necessary for avoidance of fusion with lysosomes, avoidance of other innate immune defenses, and selective interaction with specific host pathways for nutrient acquisition (Hackstadt et al., 1996; Scidmore et al., 1996; Rzomp et al., 2003, 2006; Beatty, 2006; Moore et al., 2008, 2011; Ouellette and Carabeo, 2010; Ouellette et al., 2011; Kabeiseman et al., 2013; Lucas et al., 2015). Two recent studies worked toward identifying inclusion interactomes and identified new host protein-Inc interactions (Aeberhard et al., 2015; Mirrashidi et al., 2015). However, neither study demonstrated Inc-Inc interactions, and Mirrashidi et al. did not examine Inc protein interactions in the context of a chlamydial infected cell. Therefore, *how* Incs are organized in the inclusion membrane to maximize necessary contacts with the host cell is completely unknown.

We hypothesize that Incs collaboratively function to maintain the integrity of the chlamydial inclusion with small Incs organizing the chlamydial inclusion membrane and larger Incs interfacing with host cell machinery. To test this hypothesis, we need to determine protein-protein interactions within the context of the chlamydial inclusion membrane in an infected cell. This strategy will require circumventing the incompatibility of cell lysis strategies with conditions that effectively solubilize hydrophobic proteins. Therefore, for these studies, we employed a proximity labeling technique combined with the recent ability to transform *Chlamydia*. APEX2 is a mutant soybean ascorbate peroxidase that can be genetically fused to a protein of interest to biotinylate interacting and proximal proteins (≤ 20 nm) after addition of its exogenous substrate, biotin-phenol, and catalysis of the reaction with H_2_O_2_ (Rhee et al., 2013). APEX2 targets electron-rich amino acids, such as tryptophan, tyrosine, histidine, and cysteine (Rhee et al., 2013). Biotinylation of subcellular structures, such as the inclusion membrane, can be monitored by microscopy. Further, purification of proximal or interacting proteins relies on the affinity and avidity of streptavidin for biotin. For this study, we have created Inc-APEX2 constructs and used them to examine the impact of overexpression of an early Inc (IncF) on inclusion development. We also present data demonstrating our ability to solubilize the chlamydial inclusion membrane and perform a subsequent pulldown. Importantly, completion of these studies will help us to determine specific roles for Inc proteins in the biogenesis and maintenance of the chlamydial inclusion membrane.

## Materials and methods

### Organisms and cell culture

HeLa 229 cells [American Type Culture Collection (ATCC); Manassas, VA; CCL-2.1] were cultured at 37°C with 5% CO_2_ in biotin-free DMEM (HyClone/Thermo Scientific, Logan, UT) supplemented with 10% (routine culture) or 1% fetal bovine serum (experiments involving biotinylation) (FBS; HyClone) as indicated and 10 μg/ml gentamicin (Gibco-BRL/Life Technologies; Grand Island, NY). HeLa cells were used to propagate *Chlamydia trachomatis* serovar L2 (LGV 434) for purification using established protocols (Caldwell et al., 1981; Scidmore, 2005). Chlamydial titers were determined using conventional protocols to establish multiplicities of infection (m.o.i.), which are based on inclusion forming units (i.f.u.) and determined in HeLa cells as previously described (Furness et al., 1960; Scidmore, 2005). HeLa cells and density-gradient purified *C. trachomatis* strains are routinely tested for *Mycoplasma* spp.

### Creation of Inc-APEX2 constructs

All primers used in these cloning projects are listed in Table 1. pcDNA3 APEX2-NES was a gift from Alice Ting (Addgene plasmid # 49386) (Lam et al., 2015). APEX2 contains a single N-terminal FLAG tag. To generate Inc-APEX2 fusion constructs, PCR was used to introduce restriction sites. Q5 site-directed mutagenesis (NEB, Ipswich, MA) was used to remove an internal AgeI site by creating a synonymous mutation in APEX2. The PCR products were cloned in frame into the *gfp* site of pASK_GFP_mKate2_L2 (pASK_L2) (kind gift of Dr. P. Scott Hefty, Wickstrum et al., 2013) to create IncF-APEX2, IncA_TM_-APEX2, IncA_FL_-APEX2 and APEX2 only constructs (schematic of constructs found in Supplemental Figure 1) using previously described conditions (Moore et al., 2011; Ouellette et al., 2015). Final constructs were transformed into *dam*-/*dcm- E. coli*. All constructs were confirmed by sequencing (Eurofins MWG Operon, Hunstville, AL).

**Table 1:** **Primers used for construction of Inc-APEX2**.

**Primer sequence**	**Purpose**
gg**ACCGGT**ATGACAACGCCTACTCTAATCG	Cloning IncA_TM_ into vector 5'AgeI site
gggg**GGATCC**ATTAATTTCTTTTAGAGACCCAACTT	Cloning IncA_TM_ into vector 3' BamHI site
gg**ACCGGT**ATGGGAGACGTAATGATACAGAGCG	Cloning IncF into vector with 5' AgeI site
gggg**GGATCC**GCACTTATTTGTAGAAGCGAT	Cloning IncF into vector with 3' BamHI site
CCTTCGGAACCATCAAGCACCCTGCCGA	Samesense mutation to remove AgeI sites from APEX2 using Q5 mutagenesis
GTCCGCCTGTCTTCGTGCCCTTGTCAAAG	Samesense mutation to remove AgeI sites from APEX2 using Q5 mutagenesis
GGG**CGGCCG**ctattaGTCCAGGGTCAGGCG	Cloning APEX2 into vector 3' EagI site
gggg**ACCGGT**atggactacaaggatgacgacg	Cloning APEX2 into vector 5'AgeI site
gg**ACCGGT**ATGACAACGCCTACTCTAATCG	Cloning IncA into vector 5' AgeI site
gggg**GGATCC**GGAGCTTTTTGTAGAGGGTGAT	Cloning IncA into vector 3' BamHI site

*Underlined sequences denote restriction sites*.

### Chlamydial transformation

Transformations were performed as described previously (Wang et al., 2011; Mueller and Fields, 2015; Ouellette et al., 2015). Transformants were then plaque purified as described elsewhere (Matsumoto et al., 1998; Wang et al., 2011). Localization of IncF-APEX2, IncA_TM_-APEX2, or APEX2 in chlamydiae was performed by plating HeLa cells on coverslips in a 24-well plate at 1 × 10^5^ per well and infecting with the transformed strain in the presence of 2 U/mL penicillin (IncF-APEX2, IncA_TM_-APEX2) or 1 U/mL penicillin (IncA_FL_-APEX2, APEX2) and 1 μg/mL cycloheximide. Penicillin and cycloheximide were present for all experiments to preserve the integrity of our transformants and to minimize host cell background, respectively. At 7 h post-infection (hpi), varying concentrations of anhydrotetracycline (aTc) were added and samples were fixed at the indicated times in methanol for 5 min at room temperature and stained by indirect immunofluorescence.

### Indirect immunofluorescence microscopy

HeLa cells were seeded onto 12 mm coverslips 18 h before infection with appropriate Ctr serovar L2 transformant or wild type bacteria. At indicated times post-infection, following 5 min methanol fixation, coverslips were incubated with antibodies in Table 2 to visualize construct expression (mouse anti-M2-FLAG), the inclusion membrane (rabbit anti-IncA), and organisms (guinea pig anti-Ctr L2, rabbit anti-Ctr L2), followed by incubation with the appropriate secondary antibody conjugated to DyLight fluors (Jackson ImmunoResearch Laboratories; West Grove, PA). Biotinylated proteins were detected using streptavidin conjugated to Alexa Fluor 488 (Pierce/Thermo, Rockford, IL). Coverslips were mounted on glass microscope slides using Prolong Gold mounting reagent (Life Technologies). Images were acquired using an Olympus BX60 with a 40x or 60x objective and a Nikon DS-Qi1MC digital camera or Olympus Fluoview 1000 Laser Scanning Confocal Microscope (60x objective and 2x digital magnification).

**Table 2:** **Antibodies used for indirect immunofluorescence and Western blotting**.

**Antibodies**	**Source**	**Immunofluorescence**	**Western blotting**
mouse anti-FLAG M2	Sigma	X	
rabbit anti-IncA	Ted Hackstadt^a^	X	X
guinea pig anti- *C. trachomatis* L2	Ted Hackstadt^a^	X	
rabbit anti-*C. trachomatis* L2	Ted Hackstadt^a^	X	
mouse anti-α-tubulin	Cell Signaling		X
mouse anti-GM130	BD Transduction		X
mouse anti-*C. trachomatis* Hsp60	Rick Morrison^b^		X

a *Kindly provided by Ted Hackstadt, NIAID, Rocky Mountain Laboratories, Hamilton, MT*.

b *Kindly provided by Rick Morrison, University of Arkansas Medical Center, Little Rock, AR*.

### Determination of inclusion diameter

HeLa cell monolayers were infected (m.o.i. 0.8) with Ctr serovar L2 transformants encoding IncF-APEX2, IncA_TM_-APEX2, APEX2 or wild type bacteria by centrifugation at 400 × g for 15 min at room temperature (RT) in DMEM supplemented with 1% FBS containing 2 U/mL penicillin (Ctr L2 IncF-APEX2 and Ctr L2 IncA_TM_ APEX2) or 1 U/mL penicillin (Ctr L2 APEX2) and 1 μg/mL cycloheximide and then incubated at 37°C, 5% CO_2_. At 7 hpi, transformants were either induced or not with 0.1 or 5 nM aTc. After 36 hpi, cells were fixed as above and coverslips were processed for indirect immunofluorescence. Inclusion diameter was measured using NIS elements Basic Research version 3.22 software (Nikon). Data shown are representative of two independent experiments where a minimum of 100 inclusions/coverslip were evaluated. Inclusion diameter mean and standard error of the mean were graphed using GraphPad Prism 7.0a. A one-way ANOVA with Tukey's multiple comparisons test was performed to test for statistical significance.

### Determination of plasmid retention

HeLa cells were infected with Ctr L2 transformants encoding IncF-APEX2, IncA_TM_-APEX2 or APEX2 as above. Expression of the APEX2 fusion proteins (or APEX2 alone) was induced for expression or not in the transformants with 0.1, 1, or 5 nM aTc in duplicate at 7 hpi. To visualize inclusion formation, at 24 hpi one replicate was fixed and processed for immunofluorescence to detect construct expression, chlamydial inclusions, and organisms. Infectious progeny produced were harvested at 24 hpi from duplicate wells, centrifuged 20,000 × g for 30 min, and serial dilutions in sucrose phosphate (2SP) buffer were re-infected onto HeLa cells in duplicate, essentially as previously described (Kabeiseman et al., 2013; Lucas et al., 2015). Cells were incubated at 37°C, 5% CO_2_ for 15 min, then 2SP was replaced with DMEM + 1% FBS containing 2 U/mL penicillin (Ctr L2 IncF-APEX2 and IncA_TM_-APEX2) or 1 U/mL penicillin (Ctr L2 APEX2 only) and 1 μg/mL cycloheximide. At 24 h post-secondary infection, cells were fixed in methanol and processed for indirect immunofluorescence with rabbit or guinea pig anti-Ctr L2 to distinguish aberrant vs. normal organisms. A minimum of 100 inclusions was counted per coverslip and averaged. Three individual experiments were performed for each dataset, except for 1 nM aTc induction, which was performed as two separate experiments only. The percent of inclusions containing aberrant organisms out of total number of inclusions counted was plotted using GraphPad Prism ver. 7.0a and reported as mean and standard error of the mean. A two-way ANOVA with Tukey's multiple comparisons post-test was performed to test for statistical significance.

### Live cell imaging

HeLa cells were infected with Ctr L2 transformants encoding IncF-APEX2, IncA_TM_-APEX2 as above. Transformants were either induced or not with 0.1 or 5 nM aTc at 7 hpi. 1.5 h prior to imaging, cells were labeled with 1 μM 6-((N-(7-nitrobenz-2-oxa-1, 3-diazol-4-yl)amino)hexanoyl)sphingosine (C6-NBD-ceramide); (Life Technologies) in DMEM +0.06% BSA for 20 min at 37°C, and excess label was removed via back-exchange with DMEM containing 0.6% BSA for an hour at 37°C as described previously (Hackstadt et al., 1995; Moore, 2012; Kabeiseman et al., 2013; Lucas et al., 2015). Fluorescent live cell images were acquired by mounting the coverslips onto glass slides, as previously described (Moore, 2012), and imaging at 30-40 ms exposure times with a Nikon DS-Qi1Mc camera mounted on an Olympus BX60 fluorescent scope (60x magnification). Seven to ten fields of view were taken from duplicate coverslips in two independent experiments. The fluorescent intensity (integrated density) and area of the inclusion were determined with ImageJ v1.48 (National Institutes of Health, Bethesda, MD). Mean and standard error of the mean were calculated and graphed using GraphPad Prism 7.0a software. An ordinary one-way ANOVA with Tukey's multiple comparisons *post-hoc* test was performed to test for statistical significance.

### Labeling cells with biotin-phenol and affinity purification of biotinylated proteins

For microscopic confirmation of Inc-APEX2 constructs' ability to biotinylate the inclusion membrane, HeLa cells plated in 24-well plates were infected with Ctr L2 transformants (m.o.i. 0.8) as above in DMEM supplemented with 1% FBS. HeLa cells were grown in biotin-free media (DMEM + 1% FBS) prior to infection. Ctr L2 IncF-APEX2 and IncA_TM_-APEX2 were induced with aTc at 7 hpi. At 23.5 hpi, labeling was performed essentially as previously described (Martell et al., 2012; Lam et al., 2015), and at 24 hpi, coverslips were fixed and processed for indirect immunofluorescence.

To determine proteins that were biotinylated by IncF-APEX2, HeLa cells were seeded into a 6-well plate in DMEM + 1% FBS and allowed to grow overnight. To monitor construct expression and biotinylation, coverslips are placed in 2 out of 6 wells of the 6-well plate. The cells were infected with Ctr L2 IncF-APEX2 (m.o.i. 0.75) and induced with 0.2 nM aTc at 7 hpi. At 23–26 hpi, 150 μM clastolactacystin β-lactone (clastolactacystin) (Santa Cruz biotechnology, Dallas, TX) (Johnson et al., 2015) was added to each well, and plates were incubated at 37°C 5% CO_2_ for an additional 30 min prior to biotinylation: at 23.5–26.5 hpi, monolayers were incubated with 1.5 mM biotinyl-tryamide (aka biotin-phenol) (AdipoGen, San Diego, CA) for 30 min at 37°C and 5% CO_2_. At 24–27 hpi, the labeling process was catalyzed by the addition of 3 mM H_2_O_2_ in DPBS for 1 min at RT with gentle rocking. The reaction was quenched by 3 washes at room temperature with 10 mM sodium ascorbate, 10 mM sodium azide, and 5 mM Trolox, all in DPBS. Coverslips were removed and fixed, and remaining cells were scraped into DPBS and centrifuged at 700 × g for 10 min at 4°C, and the pelleted cells were resuspended in cell lysis buffer [50 mM TrisHCl, pH 7.4, 150 mM NaCl, 0.5% sodium deoxycholate, 10 mM sodium ascorbate (Sigma), 10 mM sodium azide (Sigma), 5 mM trolox (Acros organics), 1% sodium dodecyl sulfate (SDS), 5% TritonX-100 (Sigma Aldrich), 1X HALT protease inhibitor cocktail (Pierce/Thermo), universal nuclease (Pierce), and 150 μM clastolactacystin (Santa Cruz biotechnology, Dallas, TX). Samples were sonicated three times at 30% amplitude for 20 sec and incubated on ice for 90 min, vortexing every 15 min. Lysates were centrifuged at 14,000 × g for 10 min at 4°C. Supernatants were saved, and pellets were resuspended in the cell lysis buffer described above.

Protein concentrations were quantified prior to affinity purification using EZQ protein quantification (Life technologies, Molecular probes) and normalized. Normalized lysates were added to equilibrated streptavidin magnetic beads (Pierce) and rotated for 90 min at RT. Unbound fractions of the lysate were retained and beads were washed twice by rotating with lysis buffer containing 500 mM NaCl for 5 min at RT, followed by one wash with 2 M urea in 10 mM Tris-HCl pH 8.0 and 2 washes with RIPA buffer. Proteins were eluted from streptavidin magnetic beads by 5 min denaturation at 65°C in 2x Laemmli sample buffer containing 0.5 mM biotin (Sigma). Samples were resolved by SDS-4-20%PAGE (BioRad), followed by transfer to PVDF (0.45 μm, Thermo Scientific) and Western blotting using the indicated primary antibodies (Table 2) and appropriate secondary antibodies conjugated to IRDye 680LT, IRDye CW, or a streptavidin-IRDye 680LT conjugate (LiCor Biosciences, Lincoln, NE). PVDF membranes were imaged using Odyssey CLx and processed using Image Studio version 5.2 (LiCor Biosciences). After Western blotting, some PVDF membranes were also Coomassie stained using the following protocol. Membranes were rinsed with 100% methanol followed by one wash in Tris-buffered saline with 0.1% tween 20 (TBS-T) for 5 min at RT. Membranes were stained with Coomassie Brilliant Blue R-250 (BioRad) for 2.5 min at RT and then destained with two consecutive washes of 50:43:7, methanol:dH_2_O:acetic acid and 90:10 methanol: acetic acid, respectively. Air-dried membranes were imaged using GeneSnap software (SynGene).

### Densitometry and image production

All Western blots were analyzed using Image Studio Version 5.2 software (LiCor Biosciences). Graphed data (averages and standard deviation or standard error of the mean) were produced using GraphPad Prism 7 software (GraphPad Software, La Jolla, CA). All figures were constructed using Adobe Photoshop elements 14 and Adobe Photoshop CS5 (Adobe Systems Incorporated, San Jose, CA). Any modifications to images were limited to adjustment of color balance in fluorescent images, or brightness and contrast in Western blot images. The inclusion sizes were measured using NIS elements basic research Version 3.22 (Nikon).

## Results

### Expression of APEX2 constructs from Ctr serovar L2

**Figure 1:**
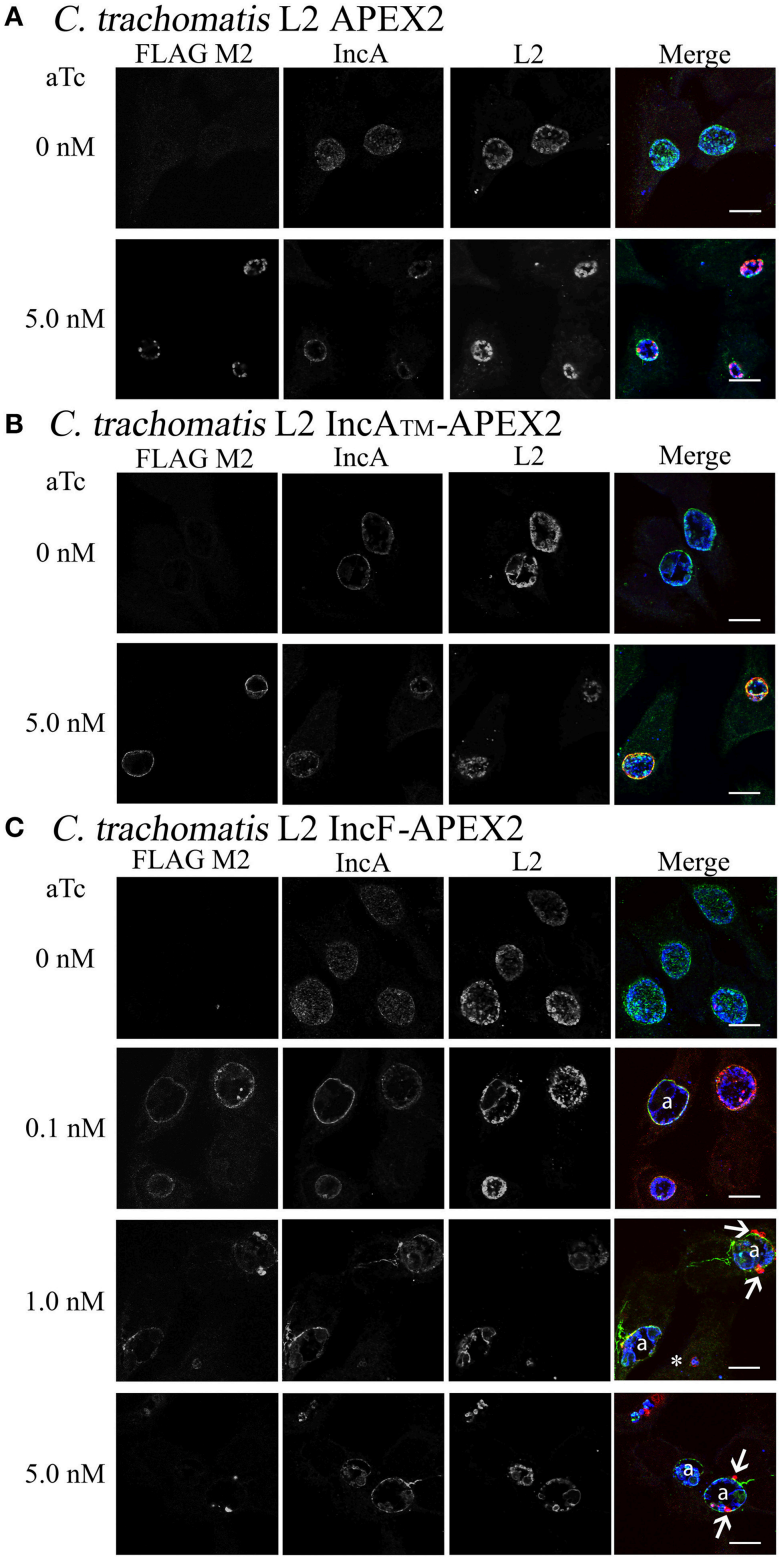
**Induction of expression of APEX2 constructs from transformed Ctr L2**. Ctr L2 was transformed with anhydrotetracycline (aTc)-inducible chlamydial expression vector pASK-L2 vector containing APEX2 **(A)**, IncA_TM_-APEX2 (TM = transmembrane domain only) **(B)**, or IncF-APEX2 **(C)**. HeLa cells were infected as described in *Materials and Methods*, and expression of constructs was induced 7 h post-infection with the indicated concentrations of aTc. Infected monolayers were fixed in methanol and processed for indirect immunofluorescence to detect expression of the construct with anti-FLAG antibody (red), the inclusion membrane with an anti-IncA antibody (green), or chlamydial organisms with an anti-Ctr L2 antibody (blue). Details for primary antibodies used in this study are found in Table 2. After incubation with the appropriate secondary antibodies, coverslips were mounted. Images were taken with Olympus Fluoview 1000 Laser Scanning Confocal Microscope (60x magnification with 2x zoom). Scale bars equal 10 μm; “a” indicates inclusions with aberrant bacteria; white arrows point to foci formed by secreted IncF-APEX2; the white asterisk indicates small inclusion formed by organisms expressing IncF-APEX2.

To better understand the function of Incs in the context of the chlamydial inclusion, we created tools that would allow the identification of interacting and proximal proteins by fusing a target Inc to APEX2 (Supplemental Figure 1). Incs are type III secreted proteins with the secretion signal located at the N-terminus; therefore, we initially verified that Inc-APEX2 constructs localized correctly to the chlamydial inclusion. In selecting which Incs to apply in these studies, we focused on IncF because of data illustrating that IncF interacts with other Inc proteins in a bacterial two-hybrid system (Gauliard et al., 2015), thus giving us the opportunity/potential to capture Inc-Inc interactions. In contrast, IncA was chosen because it is the best studied of all Incs (Rockey et al., 1997; Bannantine et al., 1998; Hackstadt et al., 1999; Delevoye et al., 2004; Suchland et al., 2008; Johnson and Fisher, 2013; Ronzone and Paumet, 2013; Weber et al., 2016), thus making it a good candidate to help develop a new system for examining both host and bacterial protein interactions at the inclusion membrane. For these studies, we transformed Ctr serovar L2 with the pASK_L2 plasmid containing either APEX2 alone, IncA_FL_-APEX2 (full length IncA), IncA_TM_-APEX2 (lacking the C-terminal cytosolic domain of IncA), and IncF-APEX2 (schematic provided in Supplemental Figure 1). HeLa cells were inoculated with the purified chlamydial transformants and expression of the constructs was induced 7 h post-infection with the indicated concentrations of anhydrotetracyline (aTc) (Figure 1 and Supplemental Figure 2). Cells were fixed at 24 h post-infection to monitor expression and localization of the construct in infected cells by immunofluorescence. Expression of the APEX2 only construct resulted in the protein remaining localized within chlamydial organisms (Figure 1A). In contrast, all Inc-APEX2 constructs localized to the chlamydial inclusion membrane (Figures 1B,C and Supplemental Figure 2). These results indicate that addition of APEX2 to the C-terminus of Inc proteins does not interfere with their type III secretion from *Chlamydia* or with their ability to localize to the chlamydial inclusion membrane.

Initially, we tested expression of our APEX2 constructs from *Chlamydia* with 5 nM aTc. However, when we examined the expression of IncF-APEX2 under these conditions, we noticed several phenomena: the formation of smaller inclusions, the development of aberrant organisms within the inclusions (presumably as a result of the chlamydial transformants losing the plasmid during the experiment), and the consolidation of IncF in small foci at the edges or outside of the inclusion. Further, “normal” sized inclusions also typically harbored aberrant *Chlamydia*, while smaller inclusions were formed by organisms expressing the IncF-APEX2 construct on the inclusion membrane (Figure 1C). Using lower concentrations of 0.1 nM aTc, IncF-APEX2 localized uniformly around the chlamydial inclusion (Figure 1C), as previously reported for endogenous IncF localization (Scidmore-Carlson et al., 1999; Shaw et al., 2000; Li et al., 2008). The exclusion of IncF-APEX2 at higher concentrations of aTc seemed unique to this construct, as IncA_FL_-APEX2 and IncA_TM_-APEX2 did not produce excluded Inc foci, even if expressed with 10 nM aTc (data not shown). Comparing IncF and IncA, IncF is expressed early in the chlamydial developmental cycle (Shaw et al., 2000) and has a small C-terminal cytosolic domain of 14 amino acids. IncA is expressed during the mid-chlamydial developmental cycle and has a much larger 193 amino acid C-terminal cytosolic domain encoding SNARE-like domains (Delevoye et al., 2008). The IncA_TM_-APEX2 construct contains only 27 amino acids of the C-terminal domain fused to APEX2 (Supplemental Figure 1); given this construct's rim-like localization to the inclusion membrane (Figure 1B), we conclude that shortened Inc C-terminal fusions to APEX2 do not compromise the localization of these constructs. In sum, these initial data indicated that overexpression of IncF is not well tolerated by chlamydiae and compromises inclusion development.

### Effect of overexpression of Inc-APEX2 constructs on inclusion development

**Figure 2:**
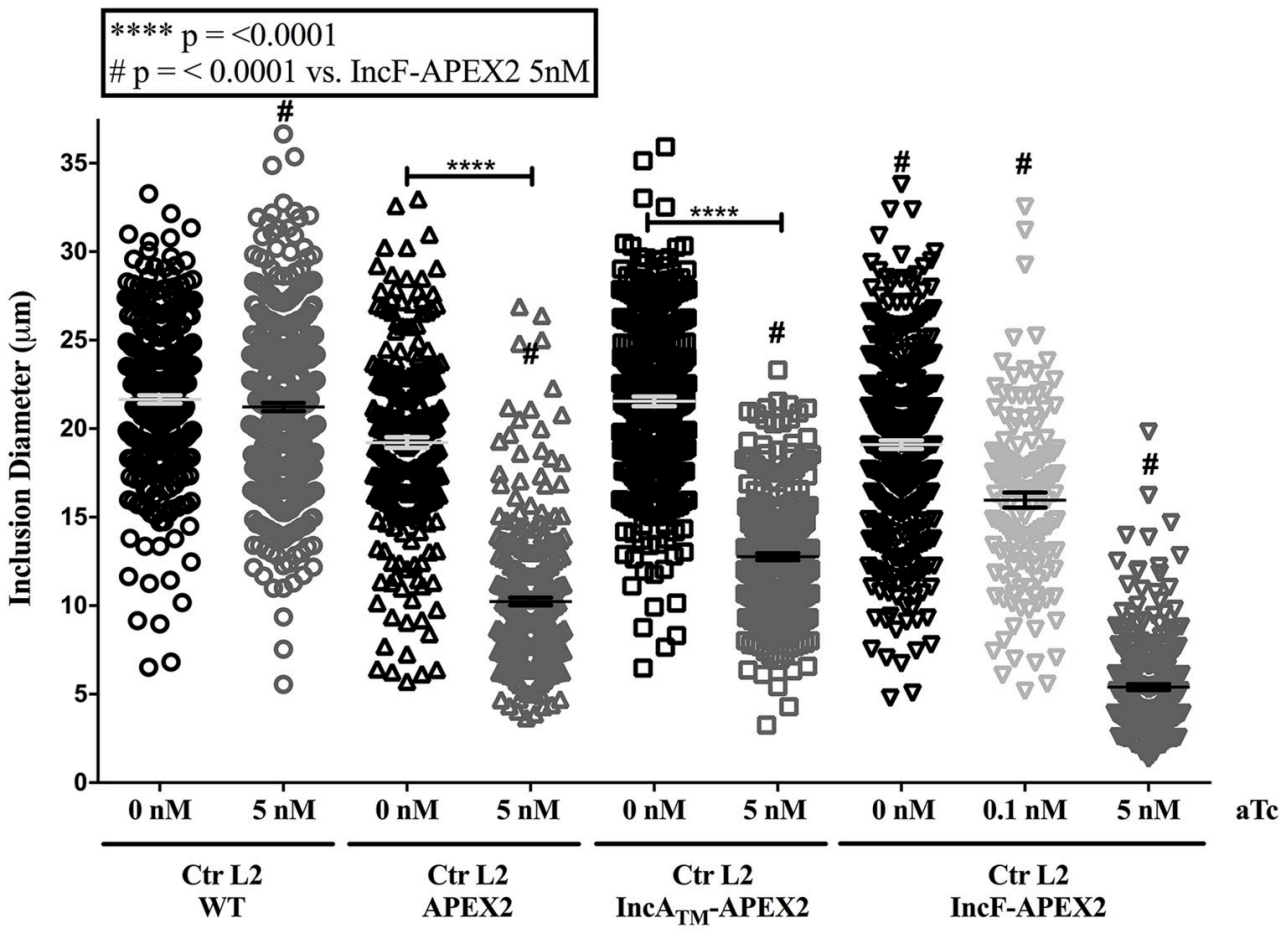
**Effect of APEX2 construct expression on inclusion diameter**. HeLa cells were infected with Ctr L2 transformants (detailed on the x-axis) as in Figure 1, and construct expression was induced with the indicated concentrations of anhydrotetracycline (aTc) at 7 h post-infection. Cells were fixed 36 h post-infection, processed for indirect immunofluorescence, and inclusion diameters (expressed in μm, y-axis) were determined as in *Materials and Methods*. Data shown include two independent experiments where 100 inclusions/coverslip were evaluated. Inclusion diameter mean and standard error of the mean are shown and were graphed using GraphPad Prism 7.0. Statistical analysis of the data included a one-way ANOVA with Tukey's multiple comparisons test; statistical significance is shown with ^****^ equaling *p* < 0.0001, and # equaling *p* < 0.0001 for all conditions specifically compared to IncF-APEX2 induced with 5 nM aTc.

To quantify the effect of Inc-APEX2 expression on chlamydial inclusion expansion, we infected HeLa cells with Ctr serovar L2 that was not transformed (wild-type, WT) or transformed with pASK_L2_APEX2, pASK_L2_IncF-APEX2, or pASK_L2_IncA_TM_-APEX2. Infected monolayers were treated with the indicated amounts of aTc at 7 h post-infection to induce the expression of the constructs. At 36 h post-infection, cells were fixed and processed for indirect immunofluorescence to detect expression of the construct, the chlamydial inclusion, and chlamydial organisms. Inclusion diameter was measured as described in *Materials and Methods* (Figure 2). Inclusions that were excluded from calculations included inclusions containing aberrant forms of *Chlamydia* and inclusions that had excluded Inc-APEX2 (see IncF-APEX2 at 5 nM aTc, Figure 1C). The diameters of inclusions formed at 36 h post-infection by untransformed Ctr serovar L2 without or with aTc were 21.65 μm ± 0.228 and 21.22 μm ± 0.231, respectively. The diameters of inclusions formed by chlamydial transformants containing pASK_L2_APEX2, pASK_L2_IncF-APEX2, or pASK_L2_IncA_TM_-APEX2 were 19.22 μm ± 0.295, 21.55 μm ± 0.267, and 19.11 μm ± 0.239, respectively. These data are consistent with transformed *Chlamydia* developing at a slightly slower rate than non-transformed strains. Induction of all constructs led to a decrease in the diameter of the inclusions, indicating an overall metabolic impact related to construct expression (Figure 2). On average, the inclusions formed by *Chlamydia* expressing APEX2 and IncA_TM_-APEX2 were statistically significantly decreased by ~9 μm, with diameters measuring 10.24 μm ± 0.218 and 12.76 μm ± 0.200, respectively. The decrease of diameter associated with expression of IncF-APEX2 was different depending on the amount of aTc used to induce expression. Induction with 0.1 nM aTc caused a statistically significant ~3.14 μm reduction in diameter (15.97 μm ± 0.431; *p* < 0.0001), while induction with 5 nM aTc caused a statistically significant ~13.8 μm decrease in inclusion diameter (5.40 μm ± 0.165). Of note, the decrease in diameter caused by expression of IncF-APEX2 with 5 nM aTc was also statistically significant from expression of APEX2 alone, IncA_TM_-APEX2 expressed with 5 nM aTc, and IncF-APEX2 expressed with 0.1 nM aTc. These data indicate that overexpression of IncF-APEX2 impairs inclusion membrane expansion above and beyond what can be attributed to a metabolic cost of producing the “exogenous” protein.

**Figure 3:**
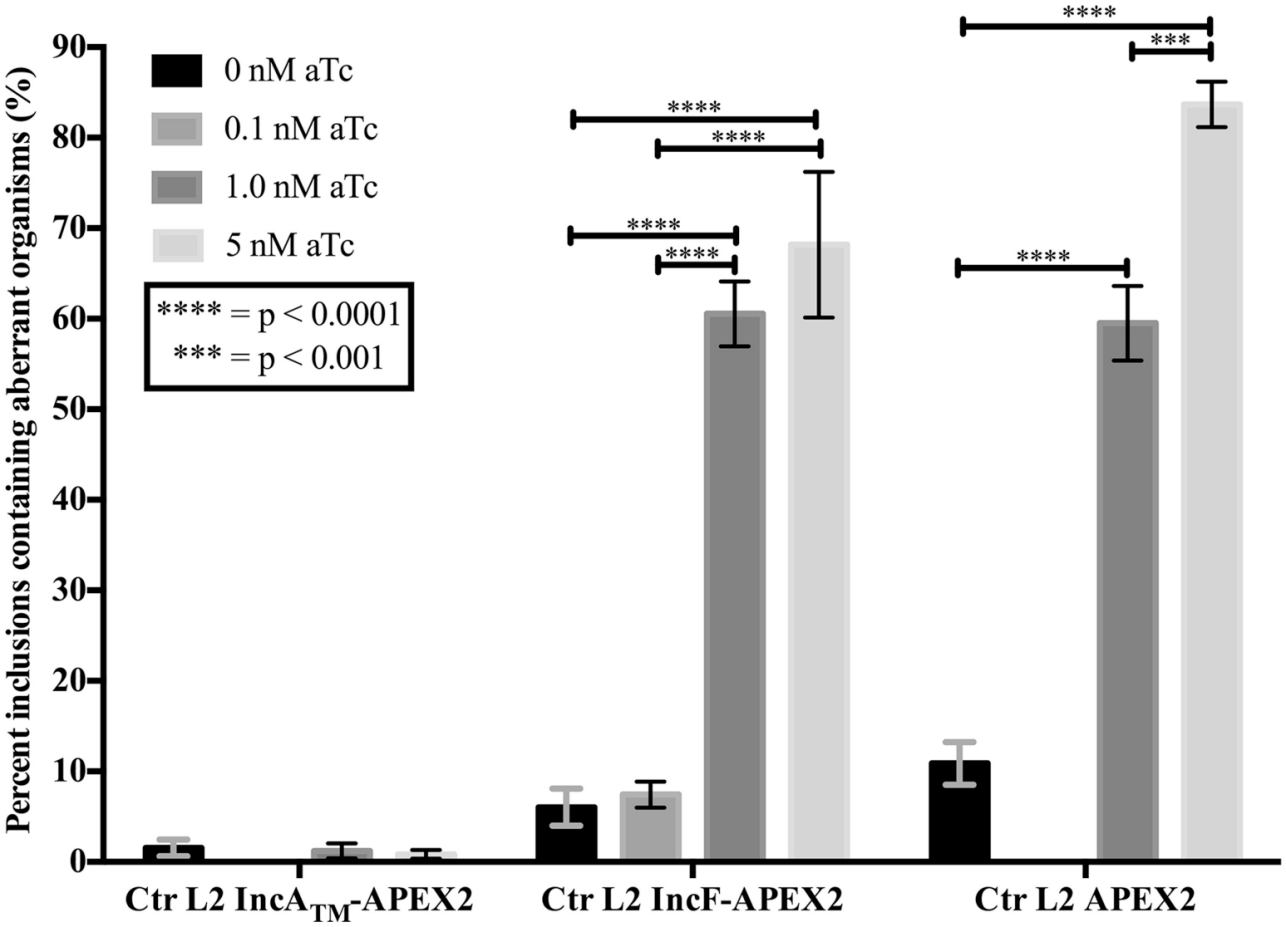
**Effect of APEX2 construct expression during the primary infection on plasmid stability**. HeLa cells were infected with Ctr L2 transformed (detailed on the x-axis) as in Figure 1, and construct expression was induced with the indicated concentrations of anhydrotetracycline (aTc) at 7 h post-infection. At 24 h post-infection duplicate coverslips were either fixed to determine construct expression or lysed to release infectious chlamydial organisms to infect a fresh monolayer (secondary infection) in the presence of penicillin to monitor retention of plasmid. *Chlamydia* that did not retain the expression plasmid would become susceptible to penicillin and become morphologically aberrant. This morphological form is readily distinguished from normal chlamydial development forms by indirect immunofluorescence microscopy. Treating Ctr L2 transformants of IncA_TM_-APEX2 or APEX2 with 0.1 nM aTc did not result in construct expression, and, therefore, these conditions were eliminated from analysis. 100 inclusions per coverslip were determined to contain either normal or aberrant *Chlamydia* (see Supplemental Figure 3). This assay had three biological replicates within two independent experiments. Data are expressed as mean and standard error of the mean of the percentage of inclusions containing aberrant organisms, were graphed, and statistically analyzed using GraphPad Prism 7.0 software. Data were analyzed for statistical significance using two-way ANOVA with Tukey's multiple comparisons post-test, and are indicated with ^****^ for *p* < 0.0001, and ^***^ for *p* < 0.001.

If overexpression of a specific construct created stress for *Chlamydia*, then one response may be for the organism to lose the plasmid and subsequently become morphologically aberrant in the presence of penicillin (the selecting agent; Supplemental Figure 3). To test for this occurrence, we infected HeLa cells with purified chlamydial transformants of IncA_TM_-APEX2, IncF-APEX2, or APEX2 alone and induced expression of the constructs with the indicated concentrations of aTc 7 h post-infection. Of note, we have found that 0.1 nM aTc does not induce detectable expression of the IncA_TM_-APEX2 or APEX2 constructs (data not shown). At 24 h post-infection, monolayers were lysed, and lysates were serially diluted and re-plated onto a fresh monolayer of HeLa cells in medium containing penicillin, which is the selectable antibiotic for the pASK-L2 plasmid (Wickstrum et al., 2013). The cells were fixed, and inclusions containing either normal or morphologically aberrant chlamydial forms were enumerated. Increasing concentrations of aTc did not increase the numbers of inclusions with aberrant forms in transformants of pASK_L2_IncA_TM_-APEX2. The incidence of plasmid loss in uninduced cultures was 1.542% ± 0.926, similar to the incidence of plasmid loss after induction of expression of the IncA_TM_-APEX2 construct with 5 nM aTc, which was 0.836 ± 0.490 (Figure 3). However, increasing concentrations of aTc did increase the numbers of inclusions bearing aberrant chlamydial forms for transformants of pASK_L2_IncF-APEX2 and pASK_L2_APEX2 (Figure 3). The incidence of plasmid loss in uninduced cultures of chlamydial transformants of pASK_L2_IncF-APEX2 was 6.042 ± 2.059%. Of note, induction of expression of IncF-APEX2 with 0.1 nM aTc, which results in correct localization of the construct to the chlamydial inclusion membrane (Figure 1C), did not cause a significant increase in incidence of plasmid loss, with 7.440 ±1.428% (Figure 3). However, at 1 nM or 5 nM aTc induction of IncF-APEX2, plasmid loss increased with 60.538 ± 3.579% and 68.182 ± 8.055% inclusions containing aberrant organisms, respectively. Plasmid loss was also noted when APEX2 was expressed with 59.508 ± 4.125% and 83.678 ± 2.519% of inclusions harboring aberrant *Chlamydia* after induction with 1 nM or 5 nM aTc, respectively. Unlike IncA_TM_-APEX2 or IncF-APEX2, which are secreted, even at high concentrations of aTc, APEX2 remains within the chlamydial organisms, thus potentially creating toxic conditions directly in the bacteria. Therefore, the increase in plasmid loss in chlamydial organisms expressing high levels of IncF-APEX2 or APEX2 only is likely creating unfavorable conditions but via different mechanisms. Of note, the aTc concentrations associated with plasmid loss in transformants expressing IncF-APEX2 were the same concentrations associated with exclusion and/or aggregation of IncF-APEX2 from the inclusion (Figure 1C) and statistically significant smaller inclusions (Figure 2).

### Effect of Inc-APEX2 expression on golgi-derived sphingomyelin acquisition

**Figure 4:**
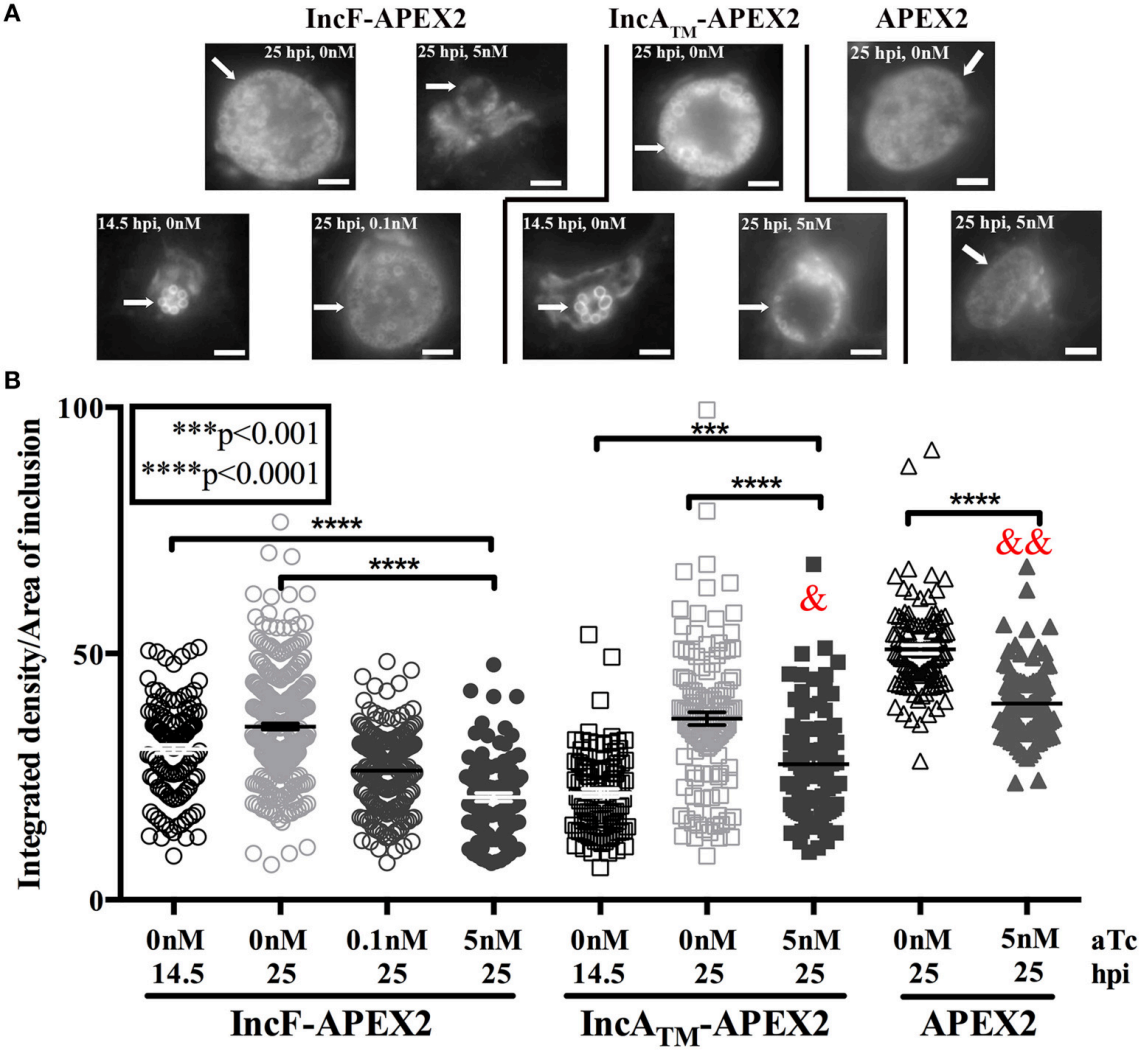
**Effect of Inc-APEX2 expression on acquisition of Golgi-derived sphingomyelin**. HeLa cells were infected with Ctr L2 transformants of IncF-APEX2, IncA_TM_-APEX2, and APEX2 only and construct expression was induced 7 h post-infection. Infected monolayers were labeled with NBD-ceramide and back-exchanged to remove fluorescent lipid trafficked to the plasma membrane as described in *Materials and Methods*. Live cell images were taken at 14.5 or 25 h post-infection using a 60x objective of an Olympus BX60 mounted with a Nikon DS-Qi1MC digital camera. Seven to ten fields of view were taken from duplicate coverslips in two independent experiments, and integrated density (brightness resulting from acquisition of fluorescent sphingomyelin) and inclusion area values were determined using ImageJ v1.48 software (National Institutes of Health, Bethesda, MD). Representative images of the matched graphed conditions are shown in **(A)**. Scale bars are equal to 5 μm, and white arrows indicate inclusions. In **(B)**, the fluorescent intensities (integrated density) divided by the areas of individual inclusions were graphed as mean and standard error of the mean using GraphPad Prism 7.0 software. Statistical significance was determined using an ordinary one-way ANOVA with Tukey's multiple comparisons post-test, with ^****^ indicating *p* < 0.0001 and ^***^ indicating *p* < 0.001. In statistical comparison of IncF-APEX2 induced with 5 nM aTc imaged at 25 hpi with indicated data points, & specifies *p* < 0.001 and && specifies *p* <0.0001.

Our combined data suggest that overexpression of IncF-APEX2 negatively impacts chlamydial health by compromising inclusion development. One metric for assessing normal inclusion development is via its interactions with host cell trafficking pathways. Chlamydiae selectively interact with exocytic vesicles, and this can be tracked via acquisition of fluorescent sphingomyelin. To ascertain if overexpression of IncF-APEX2 inhibited acquisition of fluorescent sphingomyelin, we performed live cell imaging and quantified the “brightness” per area of inclusions harboring chlamydial transformants either uninduced or induced to express IncF-APEX2, IncA_TM_-APEX2, or APEX2 only. Given the small size of IncF-APEX2 inclusions formed after induction with 5 nM aTc, we also included a comparison of inclusions formed at 14.5 h post-infection to control for effects of inclusion size vs. effects of overexpression of the construct. For these experiments, infected monolayers were labeled with NBD-ceramide then back-exchanged for 1 h prior to live-cell imaging, as described in *Materials and Methods*. As shown in the representative images in Figure 4A, 14.5 h inclusions contain only RBs, which have previously been shown to retain more sphingomyelin than inclusions with mixed RB and EB populations, as in the 25 h post-infection images (Lucas et al., 2015). Quantification of the images of uninduced samples at 14.5 and 25 h post-infection show that 14.5 h inclusions formed by Ctr transformed with pASK_L2_IncF-APEX2 or pASK_L2_IncA_TM_-APEX2 differ in “brightness” (arbitrary units of integrated density) per inclusion area (30.60 ± 0.8382 and 21.68 ± 0.8355, respectively) than 25 h inclusions formed by these same uninduced strains (35.16 ± 0.6173 and 36.79 ± 1.219) (Figure 4B). Induction of expression of IncF-APEX2 with 0.1 nM aTc resulted in slightly smaller inclusions (Figure 4A), consistent with previous results, and an overall decrease in fluorescent sphingomyelin retained by the inclusion (26.25 ± 0.5709), which was statistically significant (*p* < 0.0001) from the uninduced control at 25 hpi. However, when expression of IncF-APEX2 was induced with 5 nM aTc, sphingomyelin retention was much less (20.84 ± 0.8516), a statistically significant difference (*p* < 0.0001) from both uninduced controls at 14.5 and 25 h post-infection (Figure 4B). In comparison, 5 nM aTc induction of IncA_TM_-APEX2 or APEX2 alone resulted in slightly smaller inclusions (Figure 4A), which were “less” bright (27.51 ± 1.176 and 39.83 ± 0.6889, respectively) than the controls at 25 h post-infection (36.79 ± 1.219 and 50.87 ± 0.8435, respectively), a difference that was measured to be statistically significant (*p* < 0.0001). Of note, in comparing the amount of fluorescent label retained in inclusions induced with 5 nM aTc to express either IncF-APEX2 or IncA_TM_-APEX2, there was a statistically significant decrease between organisms in inclusions that were overexpressing IncF-APEX2 and IncA_TM_-APEX2 (20.84 ± 0.8516 vs. 27.51 ± 1.176, respectively; *p* < 0.0001), and a statistically significant decrease between organisms in inclusions that were overexpressing IncF-APEX2 and APEX2 only (20.84 ± 0.8516 vs. 39.83 ± 0.6889, *p* < 0.0001). Consistent with our previous data, overexpression of these constructs likely has a metabolic cost that negatively impacts the development of the inclusion compared to uninduced controls. Yet, these data are consistent with the notion that overexpression of specific Incs may be deleterious to inclusion formation and development. Specifically, our data indicate that, in addition to the smaller sized inclusions formed after overexpression of IncF, the inclusion is impaired in its interactions with host pathways.

### Proximity labeling of the chlamydial inclusion membrane

**Figure 5:**
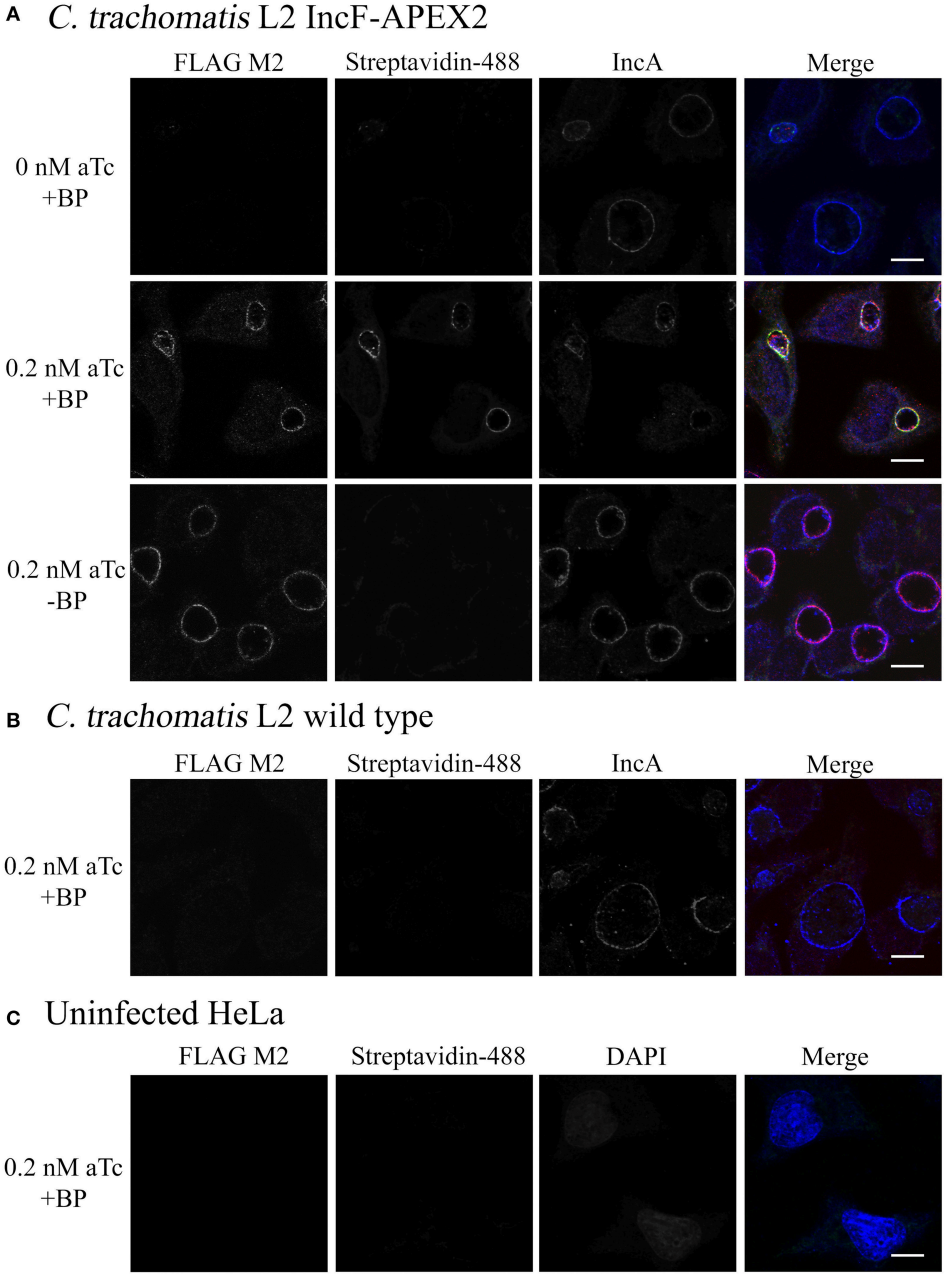
**Monitoring biotinylation of the inclusion membrane after expression of IncF-APEX2**. HeLa cells were infected with Ctr L2 transformant IncF-APEX2 **(A)**, or wild-type (not transformed) Ctr L2 **(B)** or mock infected **(C)**. Expression of IncF-APEX2 was induced using 0.2 nM aTc at 7 h post-infection. As described in *Materials and Methods*, at 27 hpi cells were labeled with biotin-phenol, fixed, and processed for indirect immunofluorescence to detect (from left to right): construct expression with an anti-FLAG antibody (red), biotinylation using streptavidin-488 (green), and the inclusion membrane using an anti-IncA antibody (blue) or DAPI (blue) to detect nuclei in uninfected cells. Negative controls (conditions not supportive of biotinylation of the inclusion membrane) in this study included Ctr transformant IncF-APEX2 not induced for expression with biotin-phenol (**A**, top row), Ctr transformant IncF-APEX2 induced with 0.2 nM aTc without biotin-phenol (**A**, bottom row), and wild-type Ctr L2 treated with both aTc and biotin-phenol **(B)**, and mock infected HeLa **(C)**. All images were taken with an Olympus Fluoview 1000 Laser Scanning Confocal Microscope (60x magnification with 2x zoom). Scale bars equal 10 μm. These images were obtained from coverslips that were removed from the 6-well plates used to produce the data in Figure 6.

The expressed goal of our study was to design a tool to allow the dynamic characterization of the inclusion membrane interactome. In the process, we found that overexpression of one of our Inc constructs negatively impacted inclusion development. While this is interesting from the perspective of learning how potential abnormalities impact chlamydial inclusion membrane organization and Inc interactions, there is a risk of not having a baseline “normal” for rigorous comparison. Therefore, we chose to optimize proximity labeling conditions with our IncF-APEX2 strain using induction conditions that did not cause significantly smaller inclusions, overt stress to the organisms (via loss of plasmid), or significant issues interacting with host cell pathways, as measured by Golgi-derived sphingomyelin retention. To test our ability to label the inclusion membrane with biotin-phenol, we infected HeLa cells with Ctr IncF-APEX2 (Figure 5A) or wild-type Ctr serovar L2 (Figure 5B). Uninfected HeLa cells were included to determine background biotinylation activities within the parameters of our experimental conditions (Figure 5C). Seven hours post-infection, we induced expression of IncF-APEX2 with concentrations of aTc (0.2 nM) found to have limited negative effects on *Chlamydia*. 26.5 h post-infection, monolayers were labeled or not with biotin-phenol for 30 min at 37°C. All monolayers were treated with H_2_O_2_, which catalyzes the labeling reaction, for an additional minute, as described in the *Materials and Methods*. The reaction was quenched and the cells were fixed in methanol and processed for immunofluorescence to detect expression of the construct, labeling of subcellular structures with biotin-phenol, and the inclusion membrane. As shown in Figure 5A, 0.2 nM aTc induces the expression of IncF-APEX2, and it was localized to the chlamydial inclusion membrane as previously seen (Figure 1). Further, addition of biotin-phenol resulted in labeling of the inclusion membrane: streptavidin-488 reacted most strongly with the inclusion membrane, and the staining colocalized with IncF-APEX2 and IncA. When no biotin-phenol is added to the medium, there is no biotinylation of the inclusion membrane or other subcellular structures in the presence of IncF-APEX2. Likewise, when IncF-APEX2 is not expressed and biotin-phenol is added to the medium, there is no labeling of the inclusion membrane or other subcellular compartments. These results were recapitulated using the IncA_TM_-APEX2 construct (Supplemental Figure 4). HeLa cells infected or not with wild-type Ctr serovar L2 treated with 0.2 nM aTc and labeled with biotin-phenol did not result in labeling of the inclusion membrane or other subcellular compartments with biotin (Figures 5B,C), indicating that our experimental conditions, as outlined in *Materials and Methods*, provide minimal to no fluorescence background in these biochemical reactions. Expression of IncF-APEX2 in combination with addition of biotin-phenol to the medium resulted in specific biotinylation of the chlamydial inclusion membrane.

### Determination of solubilization conditions for extraction of inclusion membrane associated proteins

**Figure 6:**
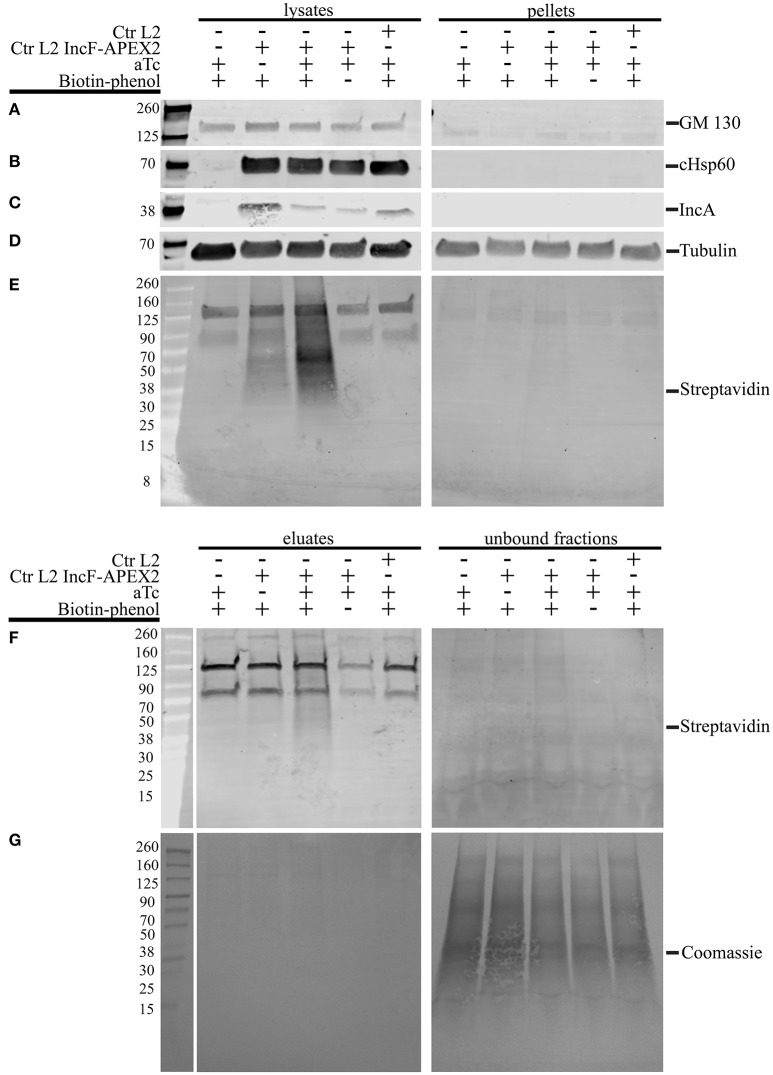
**Lysis conditions compatible with solubilization of inclusion membrane proteins and pulldown of biotinylated proteins**. HeLa cells seeded in 6-well plates were infected with Ctr L2 transformant IncF-APEX2, wild-type Ctr L2, or mock infected with the aTc induction/treatment conditions, followed by the biotin-phenol additions as indicated. The cells remaining in the 6-well plates were lysed as described in the Materials and Methods, and cleared lysates (lysates) and the insoluble pellets (pellets) were resolved by SDS-PAGE, transferred to a PVDF membrane, and Western blotted for a transmembrane Golgi protein GM130 **(A)**, chlamydial heat shock protein 60 (cHsp60) **(B)**, inclusion membrane protein IncA **(C)**, eukaryotic cytoskeletal protein tubulin **(D)**, and biotinylated proteins with streptavidin **(E)**. Cleared lysates were normalized for protein content and equal amounts of protein were added to magnetic beads conjugated to streptavidin to pull down biotinylated proteins from lysates. Western blot analysis using a streptavidin conjugate of eluate and unbound fractions from the pulldowns are shown in **(F)** and Coomassie stain of the PVDF membrane showing total protein in **(G)**.

One of the greatest difficulties in acquiring biochemical protein-protein interaction data from an inclusion is the inherent hydrophobicity of the Incs. In a separate study using proximity labeling with biotin to capture chlamydial interacting partners of eukaryotic proteins that localize to the chlamydial inclusion, we identified other eukaryotic proteins that localize to the chlamydial inclusion but failed to identify chlamydial binding partners (Rucks and Srinivasan, unpublished data). In our initial studies, we examined the biotinylation patterns from lysates representative of 5 different conditions of HeLa cells infected or not with Ctr pASK_L2_IncF-APEX2 uninduced with biotin-phenol (negative for construct expression, therefore negative for biotinylation), Ctr pASK_L2_IncF-APEX2 induced with 0.2 nM aTc with biotin-phenol (positive for construct expression and biotinylation), Ctr pASK_L2_IncF-APEX2 induced with 0.2 nM aTc without biotin-phenol (positive for construct expression, but negative for biotinylation), and Ctr serovar L2 or uninfected HeLa cells treated with 0.2 nM aTc with biotin-phenol (absolute negative controls) (as in Figure 5). IncF-APEX2 expression is detectable by indirect immunofluorescence (Figures 1, 5) but not by Western blot using either an anti-FLAG-M2 or anti-IncF antibodies (data not shown). In our initial lysis conditions, the bulk of the biotinylation was detected in the pellets of cleared supernatants (data not shown). To increase the solubility of transmembrane proteins, we increased detergent concentrations of the lysis buffer. We prepared lysates from the 5 conditions described above, and as described in the *Materials and Methods*, and blotted for (i) GM130, a eukaryotic transmembrane Golgi protein (Figure 6A), (ii) chlamydial Hsp60, a cytosolic chlamydial protein (Figure 6B), (iii) IncA, a chlamydial inclusion membrane protein (Figure 6C), (iv) tubulin, a eukaryotic cytoskeletal protein (Figure 6D), and (v) streptavidin, to monitor biotinylation within the samples (Figure 6E). Of note, the eukaryotic and chlamydial transmembrane proteins were detected within the cleared lysates but not within the pellets (Figures 6A,C). Further, the streptavidin detected the bulk of the biotinylated proteins within the cleared lysates from HeLa cells infected with Ctr pASK_L2_IncF-APEX2 induced with 0.2 nM aTc with biotin-phenol (Figure 6E, lane 3). We did detect some biotinylation within uninduced samples from HeLa cells infected with Ctr pASK_L2_IncF-APEX2 when treated with biotin-phenol but detected no biotinylation from HeLa cells infected with Ctr serovar L2, indicating that the background biotinylation is likely from leaky expression of the construct and not inherent background from the organisms (Figure 6E). Importantly, these data indicate that we have determined lysis conditions capable of solubilizing membrane proteins and, specifically, proteins within the chlamydial inclusion membrane.

To confirm that the increased detergents did not interfere with the pulldown of biotinylated proteins, we incubated these lysates with magnetic streptavidin beads and resolved the resulting eluates and unbound fractions by Western blot. As shown in Figure 6F, most of the biotinylated proteins were detected in the eluate fraction of HeLa cells infected with Ctr pASK_L2_IncF-APEX2 induced with 0.2 nM aTc with biotin-phenol. Importantly, very few biotinylated proteins were detected in the unbound fraction of this sample, despite a significant number of proteins transferred to the membrane, as indicated by Coomassie Blue staining (Figure 6G). Of note, we also detected some biotinylated proteins in the eluate fraction of HeLa cells infected with Ctr pASK_L2_IncF-APEX2 not induced with aTc but incubated with biotin-phenol. These results are consistent with the background noted in the lysates of these samples and likely due to leaky expression of the construct, as this similar pattern is not observed in eluates of untransformed Ctr. In all lysate and eluate fractions, we observed reaction of the streptavidin conjugate with a 70 and 125 kDa protein band (Figures 6E,F). Based on other studies in the laboratory, these contaminants are likely from HeLa cells, as we see them in unlabeled, uninfected HeLa cells. Consistent with these observations are other studies identifying endogenous eukaryotic biotin-binding proteins, which include pyruvate carboxylase (~125 kDa) and mitochondrial 3-methylcrotonyl carboxylase (~75 kDa) (Praul et al., 1998; Tytgat et al., 2015). However, in the eluate fractions obtained from samples containing these non-specific biotin-binding proteins (lanes 1, 4, and 5, Figure 6F), these were the only two bands that appeared in the eluate fraction, indicating that the pulldown protocol is conducive toward specific binding of biotinylated proteins. In conclusion, the lysis conditions support (i) the solubilization of proteins found in the inclusion membrane and (ii) the retention of biotinylated proteins from streptavidin beads in the presence of elevated detergent concentrations. Therefore, we have successfully developed tools to answer experimental questions on the composition of, and specific interactions within, the inclusion membrane.

## Discussion

From the moment of its entry into a host cell, the chlamydial EB is enclosed in a membrane-bound vacuole. Within this vacuole, the EB differentiates into an RB, which engages transcription and translation to produce proteins that facilitate the establishment of the chlamydial intracellular niche, the inclusion. The membrane that encloses the inclusion resembles a plasma membrane initially, but the ultimate remodeling of it results in a membrane that highly resembles an exocytic Golgi vesicle (Hackstadt et al., 1995, 1996; Moore et al., 2008). After trafficking to the microtubule-organizing center, the inclusion maintains the ability to form intimate connections with the endoplasmic reticulum (Derre et al., 2011; Elwell et al., 2011) and interacts selectively with other subcellular compartments such as the slow transferrin recycling pathway (Ouellette and Carabeo, 2010). As the developmental cycle progresses and chlamydiae multiply exponentially, the inclusion membrane grows in size to accommodate the increasing numbers of organisms (Ward, 1988). This process requires new lipids for the membrane while maintaining the ability of the inclusion to evade intracellular host defenses. Remarkably, the recruitment of host cell resources occurs across the inclusion membrane. A vast array of host proteins has been found to localize to the chlamydial inclusion, including Rab GTPases (Rzomp et al., 2003, 2006; Rejman Lipinski et al., 2009; Capmany and Damiani, 2010), which are important signaling proteins in vesicular trafficking, SNAREs (Moore et al., 2011; Kabeiseman et al., 2013; Lucas et al., 2015), which are important for membrane fusion events, and host lipid metabolism proteins (Cox et al., 2012, 2016; Recuero-Checa et al., 2016), which are important for the synthesis of lipids within the inclusion.

We have previously proposed that the inclusion acts as a pathogen-specified parasitic organelle, as increasing numbers of reports indicate that there is trafficking between the host cell and the chlamydial inclusion that may limit the impact of the chlamydial organisms on the host cell (Moore and Ouellette, 2014). As key chlamydial proteins at the host-pathogen interface, Incs make up 6% of a highly reduced genome, highlighting their importance to chlamydial fitness. Given that, over the course of the developmental cycle, *Chlamydia* shifts its nutritional sources (Ouellette and Carabeo, 2010), these shifts in nutrient acquisition may be the result of turnover or insertion of key Inc proteins in the inclusion membrane (Ouellette et al., 2011). To understand basic properties of the chlamydial inclusion, it is thus essential to acquire a greater understanding of Inc proteins. However, many questions remain about the basic properties of the chlamydial inclusion due to the difficulty in purifying to homogeneity this fragile compartment. Which Incs are absolutely required for inclusion membrane establishment, expansion, and maintenance remain unknown.

Based on data in the field, we hypothesize that Incs function collaboratively to maintain the integrity and establishment of the chlamydial inclusion. In this context, small Incs organize the chlamydial inclusion membrane and larger Incs interface with host cell machinery. The structure of Incs includes an N-terminus, which contains the type III secretion signal sequence and is typically less than 40 amino acids in length, followed by at least two transmembrane domains and a C-terminus, which ranges in size from 14 to 300 amino acids. These C-terminal portions of Incs are hypothesized to be exposed to the host cytosol (Rockey et al., 1997; Bannantine et al., 1998), although the orientation of most Incs has not been specifically examined. Implicit in our hypothesis is the assumption that Inc cytosolic domains of less than 30 amino acids are too small to favor interaction with or recruitment of host proteins. Rather, these small Incs would favor interaction with neighboring Incs in the context of the inclusion membrane. Other biological membranes contain membrane proteins that form scaffolds to support other protein-protein interactions, so-called membrane microdomains. There are reports to suggest that Inc proteins may form membrane microdomains within the inclusion membrane (Alzhanov et al., 2009; Mital et al., 2010, 2013; Weber et al., 2015). In the context of one membrane microdomain, as defined by IncB (CT232), CT101, CT222, and CT850 (Mital et al., 2010), the C-terminal domains of these Incs consist of 24, 14, 38, and 290 amino acids, respectively. Therefore, consistent with our hypothesis, IncB and CT101 may form scaffolding to stabilize CT850 and CT222 in the inclusion membrane to facilitate their interaction with host proteins. A separate study examining Inc-Inc interactions in a bacterial two-hybrid model (BACTH) demonstrated that IncF, a small Inc, can homodimerize and also associate with multiple different Incs (Gauliard et al., 2015). Challenging our assumption that small Incs primarily interact with one another are recent data demonstrating membrane microdomains formed by IncC, CT223, CT224, and CT288, which are all Inc proteins with large C-terminal domains that colocalized with phosphorylated Src (Weber et al., 2015). Until this study, there have been no tools to directly test our hypothesis or to examine specific protein-protein interactions and how proteins are organized in the inclusion membrane throughout the chlamydial developmental cycle.

To study inclusion membrane organization, we developed a number of inducibly expressed fusion constructs in Ctr to allow proximity labeling of neighboring membrane proteins. Specifically, these tools can be used to examine consequences of overexpression of Inc proteins and/or for mapping the chlamydial inclusion membrane. For our initial study, we designed Inc-APEX2 translational fusions for IncF, IncA, and IncA_TM_, where the C-terminal cytosolic domain of IncA was replaced with APEX2. IncF is expressed during the early stages of the chlamydial developmental cycle and contains a small 14 amino acid C-terminal domain, whereas IncA is expressed during the mid-stages of the chlamydial developmental cycle (Shaw et al., 2000; Gauliard et al., 2015). In our studies, both constructs localized to the chlamydial inclusion membrane after expression was induced 7 h post-infection (Figure 1). However, localization of the IncF-APEX2 construct to the inclusion membrane was highly sensitive to the amount of aTc used. Higher concentrations of aTc correlated with IncF-APEX2 aggregating outside of the chlamydial inclusion membrane (Figure 1C), significantly smaller inclusion diameter (Figure 2), loss of the plasmid harboring *incF-apex2* upon secondary infection (Figure 3), and reduced sphingomyelin trafficking to the inclusion (Figure 4). Collectively, these data indicate that overexpression of IncF is deleterious to inclusion membrane development.

A caveat to these data is that smaller inclusion diameter and loss of plasmid were noted with the Ctr strain expressing APEX2 alone after induction with 5 nM aTc, indicating that there is a metabolic burden associated with intracellular expression of APEX2. Notably, the inclusion diameters formed by organisms expressing APEX2 compared to organisms expressing IncA_TM_-APEX2 decreased on average by 20%, and this change was statistically significant (*p* < 0.0001), (Figure 2). In contrast, the inclusion diameters formed by organisms expressing IncF-APEX2 only compared to those of APEX2 or IncA_TM_-APEX2 after induction with 5 nM aTc, significantly decreased by 47.3 or 57.7%, respectively (*p* < 0.0001) (Figure 2). These comparisons are important when considering a possible connection to loss of plasmid, and thus stress of overexpression of the proteins, on the organism. 5 nM aTc induction of expression of APEX2 alone or IncF-APEX2 both resulted in loss of plasmid (Figure 3). By examining these numbers, overexpression of APEX2 alone creates a greater stress than IncF-APEX2 but likely for different reasons. APEX2 alone does not contain a signal sequence to allow the bacteria to secrete or expel the protein; hence overproduction of APEX2 may be interfering with other normal bacterial functions. Our data related to IncF-APEX2 indicate that, at 5 nM aTc induction, IncF-APEX2 is secreted but tends to aggregate outside of the chlamydial inclusion. If it remains associated with the inclusion, then the resulting diameters are incredibly small (Figures 1C, 2), suggesting that inclusion membrane expansion is inhibited and potentially creating a limiting environment for chlamydial growth and development. To confirm these interpretations, we intend to add the secretion signal of IncF to APEX2 thus allowing secretion of APEX2 once it is expressed. We anticipate in this scenario that, if APEX2 is secreted, it would be less toxic to *Chlamydia*. Secondly, we intend to perform electron micrograph analysis of the inclusions formed after induction of IncF-APEX2 to examine chlamydial developmental forms under these conditions.

In a recent study, Weber et al. induced the expression of 40 predicted Incs and localized more than half of them to the inclusion membrane (Weber et al., 2015). A caveat to this study is that a single concentration of aTc was used (10 ng/ml, which is equivalent to 20 nM) and was presumably added at the time of infection. Several of the Incs that the authors confirmed to be localized to the chlamydial inclusion are endogenously expressed during the early or middle of the chlamydial developmental cycle (specifically CT135, CT224, and CT227). Of those that did not localize to the chlamydial inclusion, induction of expression of CT616 and CT789 resulted in aberrant *Chlamydia*, presumably due to loss of the plasmid during expression (Weber et al., 2015). These results are not dissimilar to what we observed with overexpression of IncF-APEX2 with higher concentrations of aTc. Further, two of the Incs that remained associated with bacteria and did not localize to the chlamydial inclusion, CT195 and CT365, were type III secreted by the surrogate *Yersinia pseudotuberculosis* (Weber et al., 2015). Little is known about the specificity of type III secretory chaperones with their chlamydial effectors, but it is conceivable that if these effectors are expressed during later stages of the chlamydial developmental cycle, then they may not be capable of being type III secreted during the early part of the developmental cycle. The reverse would also be true: early effectors expressed later in the developmental cycle may not have the correct chaperone to mediate their secretion. In support of this, we observed that expressing IncF-APEX2 at 24 h post-infection resulted in IncF-APEX2 remaining associated with the bacteria and not localizing to the chlamydial inclusion (Supplemental Figure 5). In our study, we expressed an early Inc at 7 h post-infection, which is within the normal time of endogenous expression. We saw different negative phenotypes associated with overexpression of IncF-APEX2, particularly when overexpression of IncA_TM_-APEX2 did not cause similar negative effects on chlamydial inclusion development (Figures 2, 4) and plasmid stability (Figure 3).

In this study we have defined clear consequences on inclusion membrane development by overexpressing IncF-APEX2. We have also developed a tool to help us understand the mechanism associated with overexpression of IncF limiting the expansion of the chlamydial inclusion membrane. Before we can explore protein-protein interactions that occur after overexpression of IncF, we need to establish baseline interactions that are associated with normal inclusion development, which is why we chose to induce IncF-APEX2 with 0.2 nM aTc for the proximity labeling studies. Under these conditions, IncF-APEX2 is expressed and localized to the chlamydial inclusion membrane. Upon addition of exogenous biotin-phenol and in the presence of H_2_O_2_, the inclusion membrane is also biotinylated (Figure 5). The sample associated with the most biotinylated proteins are samples where IncF-APEX2 expression is induced in the presence of biotin-phenol (Figures 6E,F). Importantly, to identify IncF binding partners, we also created lysis conditions compatible with solubilizing known Incs (Figure 6C), and these conditions are compatible with streptavidin pulldown (Figure 6F). Ongoing studies are aimed toward identifying IncF-interaction partners with appropriate negative controls, via Western blot. A caveat to these studies is that Incs are in low abundance compared to the eukaryotic proteins, thus we are working to concentrate eluates from larger sample sizes to detect Incs. In addition, existing Inc antibodies have not performed well in our hands, for Western blot protocols, and this will require troubleshooting. We anticipate being able to confirm by mass-spectrometry analysis the ability of IncF to homodimerize within the inclusion membrane, as seen in BACTH studies (Gauliard et al., 2015), and to characterize other potential IncF-binding partners via this methodology. These can then be compared to interactions identified when IncF-APEX2 is overexpressed with 5 nM aTc. Are some of these Inc proteins excluded from the inclusion membrane? Are other interactions favored? If IncF expression is blocked or reduced, then what are the consequences and how do these relate to the protein-protein interactions characterized by the use of our APEX2 constructs? For the first time in *Chlamydia* biology, we will be able to address these important experimental questions and identify key proteins involved in inclusion membrane biogenesis, maintenance, and integrity.

## Author contributions

ER and SO conceived the experimental ideas, directed their completion. ER wrote the majority of the manuscript with MO writing the Materials and Methods. MO, LJ, and RS completed the experiments. SO, MO, LJ, and RS edited the manuscript.

### Conflict of interest statement

The authors declare that the research was conducted in the absence of any commercial or financial relationships that could be construed as a potential conflict of interest.

## Supplementary material

Supplementary Figure 1 **Schematic illustration of APEX2 constructs**. Graphical representation of APEX2, IncA-APEX2, IncA_TM_-APEX2, and IncF-APEX2 with amino acid positions indicated for Incs (gray), transmembrane domain (TM, white), single FLAG epitope tag (red), and APEX2 (blue). TM domains were determined using TopCons (http://topcons.cbr.su.se/pred/). The diagrams are not drawn to scale.

Supplementary Figure 2 **Inducible expression of IncA-APEX2 in Ctr L2**. HeLa cells were infected with Ctr L2 transformed with pASK_L2 containing IncA_fl_-APEX2 (fl = full length), and 7 h post-infection construct expression was induced with 5 nM anhydrotetracycline (aTc). Twenty four hour post-infection, cells were fixed and processed for indirect immunofluorescence to detect construct expression with an anti-FLAG antibody (red), inclusion membrane with an anti-IncA antibody (green), and nuclei and chlamydial organisms with DAPI (blue). Coverslips were imaged with an Olympus Fluoview 1000 Laser Scanning Confocal Microscope (60x magnification with 2x zoom). Scale bars equal 10 μm.

Supplementary Figure 3 **Comparison of normal and aberrant chlamydial forms by indirect immunofluorescence microscopy**. HeLa cells were infected with *C. trachomatis* serovar L2 or Ctr L2 transformed with pASK_L2_IncF-APEX2. IncF-APEX2 expression was induced with addition of 5 nM anhydrotetracycline (aTc) 7 h post-infection. Monolayers were fixed at 24 h post-infection and organisms were stained with an anti-Ctr L2 antibody, followed by a secondary conjugated to Alexa Fluor- 488 (WT) or 405 (IncF-APEX2). Penicillin will inhibit chlamydial cell division and is used as the selecting agent for transformation; organisms containing the plasmid will be insensitive to penicillin treatment. Monolayers infected with *C. trachomatis* serovar L2 without the addition of penicillin show inclusions with typical chlamydial developmental forms (≤ 1 μm). In contrast, monolayers infected with *C. trachomatis* serovar L2 and treated with penicillin show enlarged, aberrant development forms, consistent with a blockage in cell division. Similar aberrant developmental forms are noted after expression of IncF-APEX2 with 5 nM aTc; in these images, the transformants have lost the plasmid in response to overexpression of IncF-APEX2. Select aberrant chlamydial forms are denoted with white arrows. Scale bars equal 10 μm.

Supplementary Figure 4 **Biotin labeling of inclusion membrane by Ctr L2 IncA_TM_-APEX2**. HeLa cells were seeded on coverslips and infected with Ctr L2 IncA_TM_-APEX2 transformant. 7 hpi, cells were either uninduced or induced with 5 nM aTc. 40 h post-infection, cells were either labeled for 30 min with biotin-phenol (BP) or not, then treated with H_2_O_2_ to catalyze biotinylation of neighboring proteins. Cells were fixed in methanol and processed for immunofluorescence to detect IncA_TM_-APEX2 (red), biotinylation (green), and the inclusion and nuclei (blue).

Supplementary Figure 5 **Induction of IncF-APEX2 at different time points post-infection with Ctr L2**. HeLa cells were infected with Ctr L2 IncF-APEX2 transformants, and 5 nM anhydrotetracycline (aTc) was used to induce expression of the construct at 3, 7, 12, 24, and 30 h post-infection. Cells were fixed and processed for indirect immunofluorescence to detect the expression of construct with an anti-FLAG antibody (red) or the inclusion membrane with an anti-IncA antibody (green). Images were taken at 40X magnification with an Olympus BX60 mounted with a Nikon DS-Qi1MC digital camera. Scale bars equal 10 μm.
