# Supplementary figures and images for: Development of a Proximity Labeling System to Map the Chlamydia trachomatis Inclusion Membrane
Source: Front Cell Infect Microbiol. 2017 Feb 15;7:40. doi: 10.3389/fcimb.2017.00040 (PMC5309262; doi:10.3389/fcimb.2017.00040)

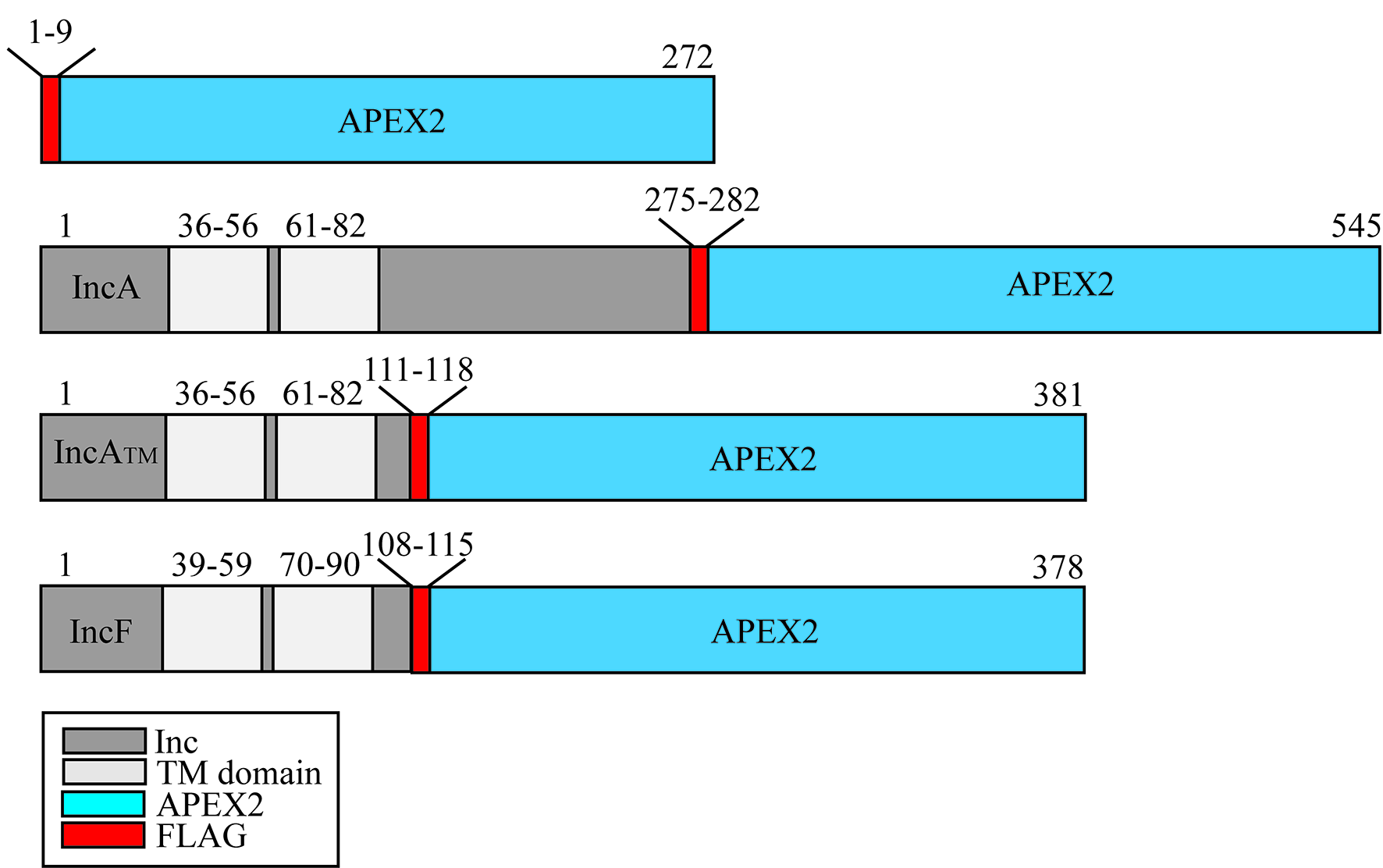

Supplement: Supplementary Figure 1 — Schematic illustration of APEX2 constructs. Graphical representation of APEX2, IncA-APEX2, IncATM-APEX2, and IncF-APEX2 with amino acid positions indicated for Incs (gray), transmembrane domain (TM, white), single FLAG epitope tag (red), and APEX2 (blue). TM domains were determined using TopCons (http://topcons.cbr.su.se/pred/). The diagrams are not drawn to scale. [file Image1.TIF]

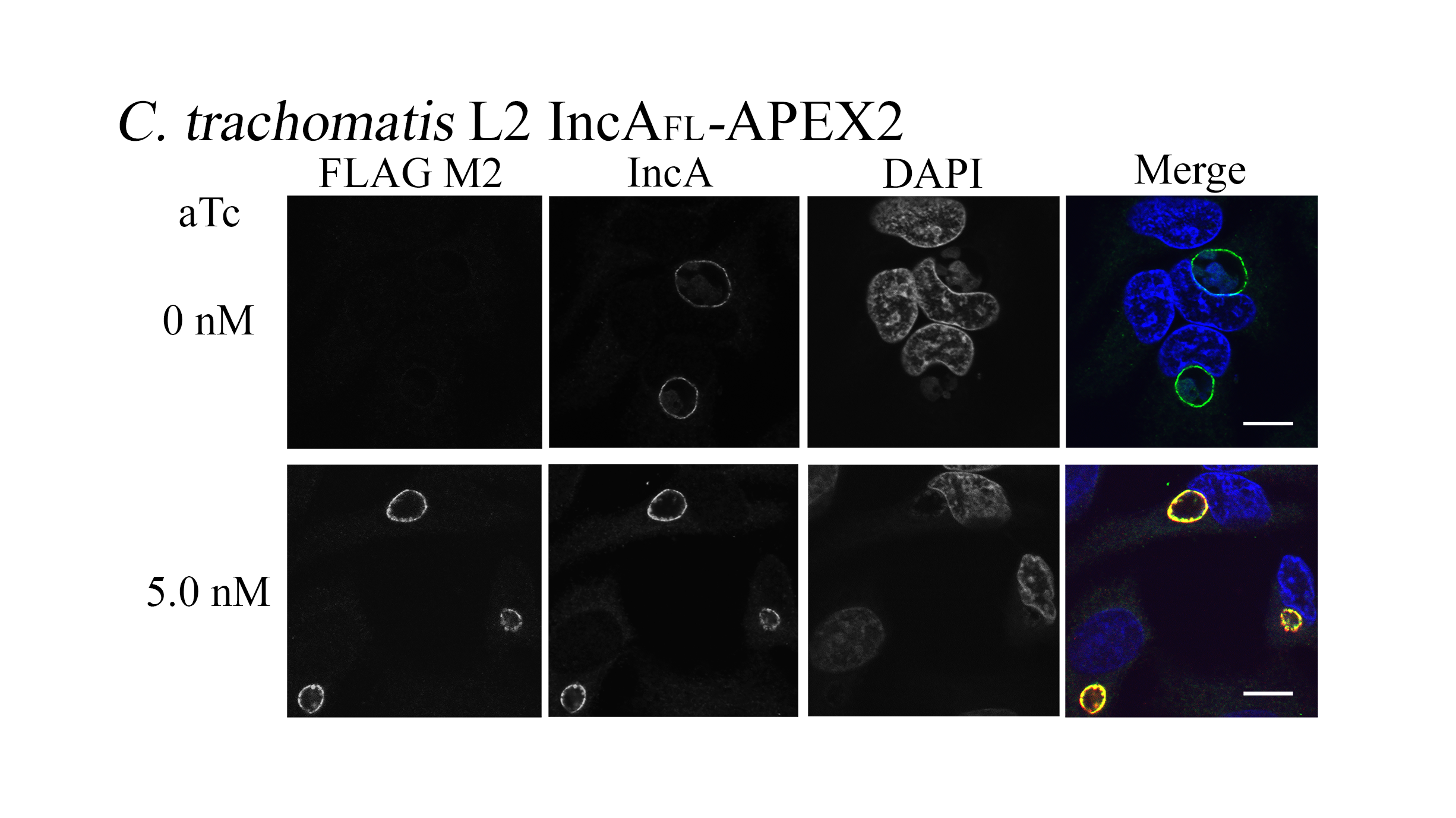

Supplement: Supplementary Figure 2 — Inducible expression of IncA-APEX2 in Ctr L2. HeLa cells were infected with Ctr L2 transformed with pASK_L2 containing IncAfl-APEX2 (fl = full length), and 7 h post-infection construct expression was induced with 5 nM anhydrotetracycline (aTc). Twenty four hour post-infection, cells were fixed and processed for indirect immunofluorescence to detect construct expression with an anti-FLAG antibody (red), inclusion membrane with an anti-IncA antibody (green), and nuclei and chlamydial organisms with DAPI (blue). Coverslips were imaged with an Olympus Fluoview 1000 Laser Scanning Confocal Microscope (60x magnification with 2x zoom). Scale bars equal 10 μm. [file Image2.TIF]

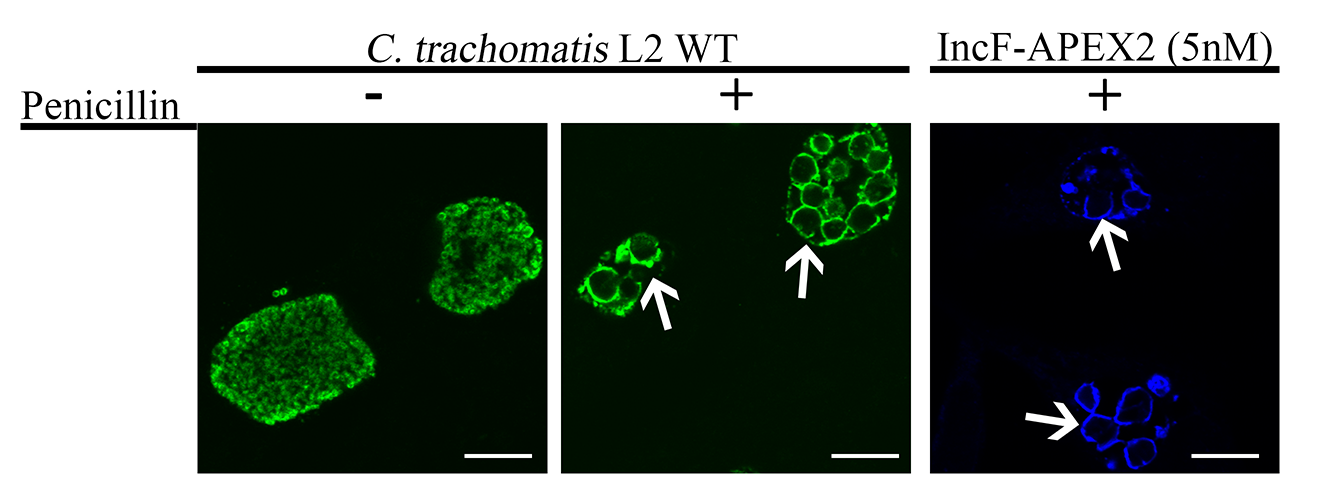

Supplement: Supplementary Figure 3 — Comparison of normal and aberrant chlamydial forms by indirect immunofluorescence microscopy. HeLa cells were infected with C. trachomatis serovar L2 or Ctr L2 transformed with pASK_L2_IncF-APEX2. IncF-APEX2 expression was induced with addition of 5 nM anhydrotetracycline (aTc) 7 h post-infection. Monolayers were fixed at 24 h post-infection and organisms were stained with an anti-Ctr L2 antibody, followed by a secondary conjugated to Alexa Fluor- 488 (WT) or 405 (IncF-APEX2). Penicillin will inhibit chlamydial cell division and is used as the selecting agent for transformation; organisms containing the plasmid will be insensitive to penicillin treatment. Monolayers infected with C. trachomatis serovar L2 without the addition of penicillin show inclusions with typical chlamydial developmental forms (≤ 1 μm). In contrast, monolayers infected with C. trachomatis serovar L2 and treated with penicillin show enlarged, aberrant development forms, consistent with a blockage in cell division. Similar aberrant developmental forms are noted after expression of IncF-APEX2 with 5 nM aTc; in these images, the transformants have lost the plasmid in response to overexpression of IncF-APEX2. Select aberrant chlamydial forms are denoted with white arrows. Scale bars equal 10 μm. [file Image3.TIF]

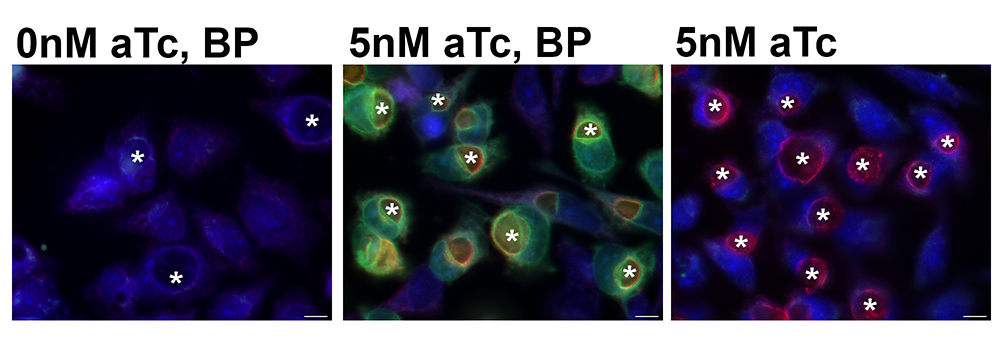

Supplement: Supplementary Figure 4 — Biotin labeling of inclusion membrane by Ctr L2 IncATM-APEX2. HeLa cells were seeded on coverslips and infected with Ctr L2 IncATM-APEX2 transformant. 7 hpi, cells were either uninduced or induced with 5 nM aTc. 40 h post-infection, cells were either labeled for 30 min with biotin-phenol (BP) or not, then treated with H2O2 to catalyze biotinylation of neighboring proteins. Cells were fixed in methanol and processed for immunofluorescence to detect IncATM-APEX2 (red), biotinylation (green), and the inclusion and nuclei (blue). [file Image4.TIF]

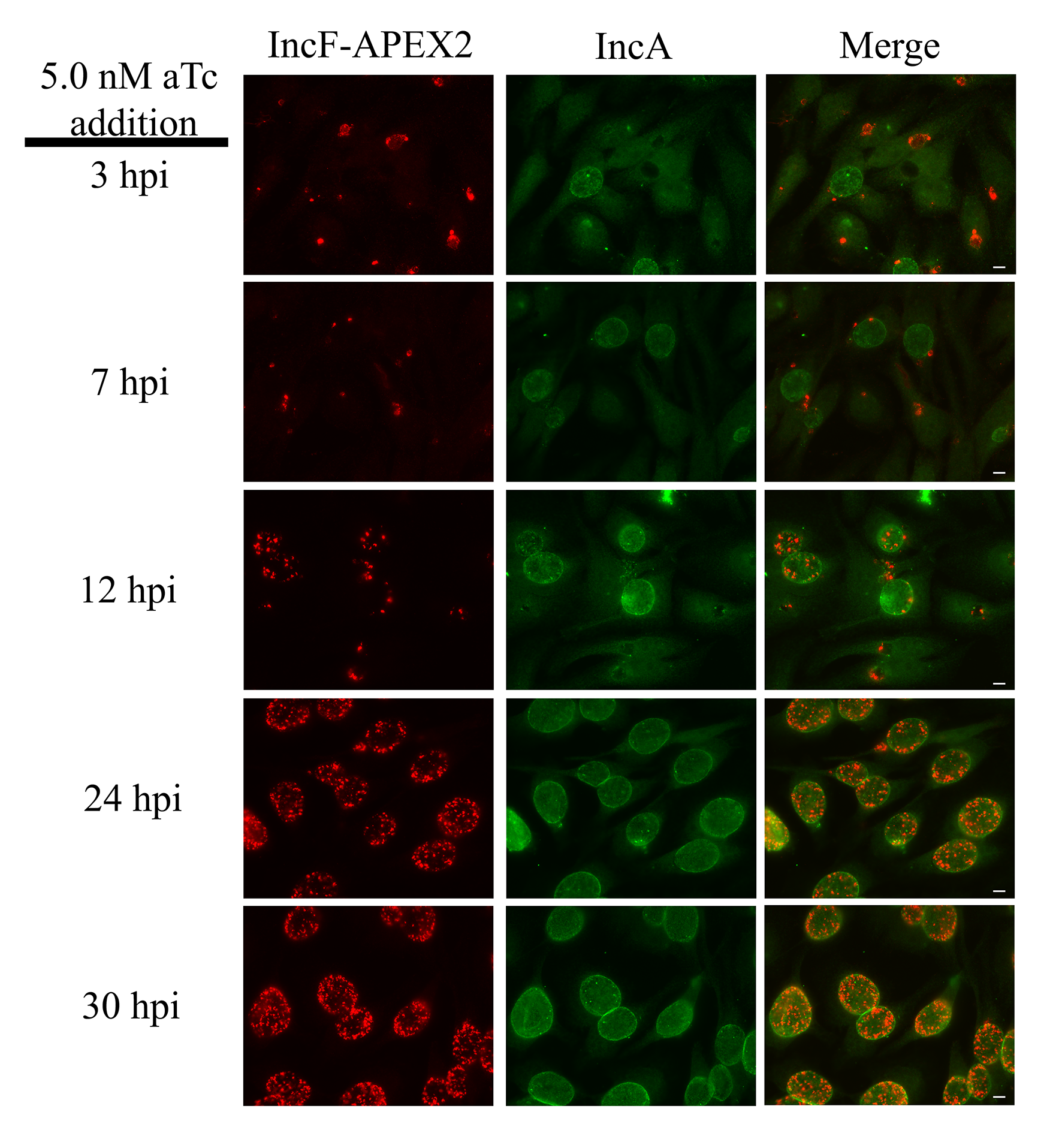

Supplement: Supplementary Figure 5 — Induction of IncF-APEX2 at different time points post-infection with Ctr L2. HeLa cells were infected with Ctr L2 IncF-APEX2 transformants, and 5 nM anhydrotetracycline (aTc) was used to induce expression of the construct at 3, 7, 12, 24, and 30 h post-infection. Cells were fixed and processed for indirect immunofluorescence to detect the expression of construct with an anti-FLAG antibody (red) or the inclusion membrane with an anti-IncA antibody (green). Images were taken at 40X magnification with an Olympus BX60 mounted with a Nikon DS-Qi1MC digital camera. Scale bars equal 10 μm. [file Image5.TIF]
